# Chloride deregulation and GABA depolarization in MTOR-related malformations of cortical development

**DOI:** 10.1093/brain/awae262

**Published:** 2024-08-06

**Authors:** Naziha Bakouh, Reyes Castaño-Martín, Alice Metais, Emanuela Loredana Dan, Estelle Balducci, Cerina Chhuon, Joanna Lepicka, Giulia Barcia, Emma Losito, Stéphane Lourdel, Gabrielle Planelles, Raul C Muresan, Vasile Vlad Moca, Anna Kaminska, Marie Bourgeois, Nicole Chemaly, Yasmine Rguez, Stéphane Auvin, Gilles Huberfeld, Pascale Varlet, Vahid Asnafi, Ida Chiara Guerrera, Edor Kabashi, Rima Nabbout, Sorana Ciura, Thomas Blauwblomme

**Affiliations:** Translational Research in Neuroscience Lab, Institut Imagine, Université Paris Cité, INSERM U1163, 75015 Paris, France; Translational Research in Neuroscience Lab, Institut Imagine, Université Paris Cité, INSERM U1163, 75015 Paris, France; Institute of Psychiatry and Neuroscience of Paris (IPNP), Université Paris Cité, INSERM U1266, 75014 Paris, France; Service de Neuropathologie, GHU-Paris Psychiatrie et Neurosciences, Hôpital Sainte Anne, F-75014 Paris, France; STAR-UBB Institute, Babeş-Bolyai University, 400084 Cluj-Napoca, Romania; Department of Pediatric Neurosurgery Hôpital Necker, Assistance Publique Hôpitaux de Paris, 75015 Paris, France; Department of Pediatric Neurology, Hôpital Necker, Assistance Publique Hôpitaux de Paris, 75015 Paris, France; INSERM US24, Proteomic platform, SFR Necker, 75015 Paris, France; INSERM US24, Proteomic platform, SFR Necker, 75015 Paris, France; Translational Research in Neuroscience Lab, Institut Imagine, Université Paris Cité, INSERM U1163, 75015 Paris, France; Department of Pediatric Neurosurgery Hôpital Necker, Assistance Publique Hôpitaux de Paris, 75015 Paris, France; Department of Pediatric Neurology, Hôpital Necker, Assistance Publique Hôpitaux de Paris, 75015 Paris, France; Department of Pediatric Neurosurgery Hôpital Necker, Assistance Publique Hôpitaux de Paris, 75015 Paris, France; Department of Pediatric Neurology, Hôpital Necker, Assistance Publique Hôpitaux de Paris, 75015 Paris, France; Cordeliers Research Center, INSERM, Sorbonne University, Paris Cité University, 75006 Paris, France; CNRS EMR 8228—Laboratory of Renal Physiology and Tubulopathies, Université de Paris Cité, Centre de Recherche des Cordeliers, 75006 Paris, France; Cordeliers Research Center, INSERM, Sorbonne University, Paris Cité University, 75006 Paris, France; CNRS EMR 8228—Laboratory of Renal Physiology and Tubulopathies, Université de Paris Cité, Centre de Recherche des Cordeliers, 75006 Paris, France; STAR-UBB Institute, Babeş-Bolyai University, 400084 Cluj-Napoca, Romania; STAR-UBB Institute, Babeş-Bolyai University, 400084 Cluj-Napoca, Romania; Department of Pediatric Neurosurgery Hôpital Necker, Assistance Publique Hôpitaux de Paris, 75015 Paris, France; Department of Pediatric Neurology, Hôpital Necker, Assistance Publique Hôpitaux de Paris, 75015 Paris, France; Department of Pediatric Neurosurgery Hôpital Necker, Assistance Publique Hôpitaux de Paris, 75015 Paris, France; Department of Pediatric Neurology, Hôpital Necker, Assistance Publique Hôpitaux de Paris, 75015 Paris, France; Department of Pediatric Neurosurgery Hôpital Necker, Assistance Publique Hôpitaux de Paris, 75015 Paris, France; Department of Pediatric Neurology, Hôpital Necker, Assistance Publique Hôpitaux de Paris, 75015 Paris, France; Institute of Psychiatry and Neuroscience of Paris (IPNP), Université Paris Cité, INSERM U1266, 75014 Paris, France; Hôpital Robert Debré, Assistance Publique Hôpitaux de Paris, 75019 Paris, France; Institute of Psychiatry and Neuroscience of Paris (IPNP), Université Paris Cité, INSERM U1266, 75014 Paris, France; Institute of Psychiatry and Neuroscience of Paris (IPNP), Université Paris Cité, INSERM U1266, 75014 Paris, France; Service de Neuropathologie, GHU-Paris Psychiatrie et Neurosciences, Hôpital Sainte Anne, F-75014 Paris, France; Department of Pediatric Neurosurgery Hôpital Necker, Assistance Publique Hôpitaux de Paris, 75015 Paris, France; Department of Pediatric Neurology, Hôpital Necker, Assistance Publique Hôpitaux de Paris, 75015 Paris, France; INSERM US24, Proteomic platform, SFR Necker, 75015 Paris, France; Translational Research in Neuroscience Lab, Institut Imagine, Université Paris Cité, INSERM U1163, 75015 Paris, France; Translational Research in Neuroscience Lab, Institut Imagine, Université Paris Cité, INSERM U1163, 75015 Paris, France; Department of Pediatric Neurosurgery Hôpital Necker, Assistance Publique Hôpitaux de Paris, 75015 Paris, France; Department of Pediatric Neurology, Hôpital Necker, Assistance Publique Hôpitaux de Paris, 75015 Paris, France; Translational Research in Neuroscience Lab, Institut Imagine, Université Paris Cité, INSERM U1163, 75015 Paris, France; Translational Research in Neuroscience Lab, Institut Imagine, Université Paris Cité, INSERM U1163, 75015 Paris, France; Department of Pediatric Neurosurgery Hôpital Necker, Assistance Publique Hôpitaux de Paris, 75015 Paris, France; Department of Pediatric Neurology, Hôpital Necker, Assistance Publique Hôpitaux de Paris, 75015 Paris, France

**Keywords:** malformation of cortical dysplasia, epilepsy, GABA_A_ receptor, WNK1/SPAK-OSR1, mTOR, rapamycin

## Abstract

Focal cortical dysplasia, hemimegalencephaly and cortical tubers are paediatric epileptogenic malformations of cortical development (MCDs) frequently pharmacoresistant and mostly treated surgically by the resection of epileptic cortex. Availability of cortical resection samples has allowed significant mechanistic discoveries directly from human material. Causal brain somatic or germline mutations in the *AKT*/*PI3K*/*DEPDC5*/*MTOR* genes have been identified. GABA_A_-mediated paradoxical depolarization, related to altered chloride (Cl^−^) homeostasis, has been shown to participate to ictogenesis in human paediatric MCDs. However, the link between genomic alterations and neuronal hyperexcitability is unclear. Here, we studied the post-translational interactions between the mTOR pathway and the regulation of cation–chloride cotransporters (CCCs), KCC2 and NKCC1, that are largely responsible for controlling intracellular Cl^−^ and, ultimately, GABAergic transmission.

For this study, 35 children (25 MTORopathies and 10 pseudo-controls, diagnosed by histology plus genetic profiling) were operated for drug-resistant epilepsy. Postoperative cortical tissues were recorded on a multi-electrode array to map epileptic activities. CCC expression level and phosphorylation status of the WNK1/SPAK-OSR1 pathway was measured during basal conditions and after pharmacological modulation. Direct interactions between mTOR and WNK1 pathway components were investigated by immunoprecipitation. Membranous incorporation of MCD samples in *Xenopus laevis* oocytes enabled measurement of the Cl^−^ conductance and equilibrium potential for GABA.

Of the 25 clinical cases, half harboured a somatic mutation in the mTOR pathway, and pS6 expression was increased in all MCD samples. Spontaneous interictal discharges were recorded in 65% of the slices. CCC expression was altered in MCDs, with a reduced KCC2/NKCC1 ratio and decreased KCC2 membranous expression. CCC expression was regulated by the WNK1/SPAK-OSR1 kinases through direct phosphorylation of Thr^906^ on KCC2, which was reversed by WNK1 and SPAK antagonists (*N*-ethylmaleimide and staurosporine). The mSIN1 subunit of MTORC2 was found to interact with SPAK-OSR1 and WNK1. Interactions between these key epileptogenic pathways could be reversed by the mTOR-specific antagonist rapamycin, leading to a dephosphorylation of CCCs and recovery of the KCC2/NKCC1 ratio. The functional effect of such recovery was validated by the restoration of the depolarizing shift in the equilibrium potential for GABA by rapamycin, measured after incorporation of MCD membranes into *X. laevis* oocytes, in line with a re-establishment of normal Cl^−^ reversal potential.

Our study deciphers a protein interaction network through a phosphorylation cascade between MTOR and WNK1/SPAK-OSR1 leading to deregulation of chloride cotransporters, increased neuronal Cl^−^ levels and GABA_A_ dysfunction in malformations of cortical development, linking genomic defects and functional effects and paving the way to target epilepsy therapy.

## Teaser

Bakouh *et al*. study the mechanisms underlying epilepsy in focal cortical dysplasia, and show that somatic brain mutations in mTOR drive the deregulation of chloride co-transporters, leading to paradoxical GABA depolarization and neuronal hyperexcitability.

## Introduction

Focal cortical dysplasia (FCD) is a frequent aetiology of focal drug-resistant epilepsy requiring surgical resections early in life to treat seizures.^1,2^ Direct access to the epileptic cortex allowed its early histological description 50 years ago, the tissue harbouring ‘large bizarre neurons, grotesque cells probably of glial origin, and a global appearance reminiscent yet distinct to tuberous sclerosis’.^3^ Further clinical characterization identified among these malformations of cortical development a distinctive clinical presentation (early onset of seizures, pseudo-continuous interictal spikes on EEG, cortical T_2_ hyperintensity along a trans mantle sign and PET hypometabolism),^4-6^ associated with stereotyped histological abnormalities (loss of laminar and columnar organization, cytomegalic neurons and balloon cells) classified as FCD type IIb.^7,8^ FCD type II was further hallmarked by the discovery that a somatic mutation in the mTOR gene [(*MTOR*) c.7280T>C (p.Leu2427Pro)] was causative of the cortical disorganization and epilepsy, because *in utero* electroporation of the mutated mTOR in mouse embryos recapitulated key features of the human disease.^9^ This was later confirmed when larger cohorts identified both germline and single somatic mutation of the MTOR pathway in focal cortical dysplasia, hemimegalencephaly (HMG) and cortical tubers (TSC), including *PI3K*, *AKT*, *DEPDC5*, *TSC1-2* mutations in >80% of the samples, leading to a hyperactive MTOR pathway with high levels of expression of its downstream effector phospho-S6 (pS6).^10-13^ Common mechanisms might therefore underlie epileptogenesis in different histological entities grouped as mTORopathies or MTOR-related malformations of cortical development (MCDs). Deciphering how these mutations translate into neuronal hyperexcitability and clinical seizures is, however, hampered by the low level of variant allele frequency (<5%) in the case of somatic mutations and by the role of MTOR as a critical hub in many cellular signalling pathways (autophagy, protein synthesis and cell survival).^13,14^ At the transcriptional level, *AKT* mutation can lead through FOXG1 transcription factor to Reelin derepression and paracrine Reelin secretion causing neuronal altered migration.^15^ MTOR-related RNA translational deregulation increases the expression of IRSp53, CREB1 and ADK, which are associated with epileptic seizures.^16^ These molecular studies do not account definitively for the mechanisms underlying seizures in the dysplastic cortex, where imbalance between inhibitory and excitatory networks sustains epileptic activity.^17-19^ GABAergic transmission is particularly deregulated in FCD, with a global reduction of the number of interneurons that cluster around cytomegalic cells at the junction of grey and white matter.^20,21^ GABA_A_ neurotransmission has been shown to be positively involved in seizure initiation in *in vitro* preparations of human MCD cortical slices^22^ and is associated with a GABA_A_-dependent pacemaker activity of dystrophic and cytomegalic neurons.^23-25^ This paradoxical GABAergic depolarization might be explained by neuronal chloride accumulation leading to a shift in its reversal potential owing to altered expression of chloride neuronal cotransporters. Specifically, decreased levels of the membrane chloride extruder KCC2 (SLC12A5) and abnormal expression of the chloride intruder NKCC1 (SLC12A2) were observed in various FCD subtypes.^26-32^ The expression of NKCC1 and KCC2 is regulated, in part, through post-translational phosphorylation cascades associated with the WNK1/SPAK-OSR1 pathway: WNK1 activates the kinases SPAK and OSR1, which directly phosphorylate and stimulate the expression of chloride intruder NKCC1, while inhibiting the expression of chloride extruder KCC2 through declustering and endocytosis.^33,34^ Interestingly, the WNK pathway is regulated upstream by the PI3/AKT/mTOR pathway, because AKT directly phosphorylates WNK1 and indirectly activates WNK3-WNK4, and mTORC2 directly phosphorylates OSR1.^35,36^ The link between the mTOR mutations and epileptogenicity have been addressed mainly from an architectural point of view, with defective neuronal migration and dysmorphogenesis,^15,37^ but the link between genetic alterations and GABA_A_-dependent epileptogenesis remains to be addressed.

Here, we postulate that mTOR pathway mutation can deregulate chloride cotransporter at a post-translational level, leading to a shift in Cl^−^ reversal potential (*E*_Cl_) and paradoxical GABA_A_-induced depolarization in MCD. To address this, we performed *ex vivo* electrophysiology and biochemistry studies on human cortical slices from FCD, HMG and TSC, grouped as MTOR-related MCDs. We report direct interactions between mTOR and WNK1/SPAK-OSR1/NKCC1/KCC2 that sustain GABAergically mediated hyperexcitability in MCDs and could be targeted pharmacologically to treat seizures.

## Materials and methods

### Patients and study design

The parents or legal guardians of patients gave informed written consent for the storage and research on biological specimens provided as part of treatment. The biological collection stored in our institution and the study are declared under numbers D2009-955 and ANR 20-CE17-003, respectively.

**Figure 1:**
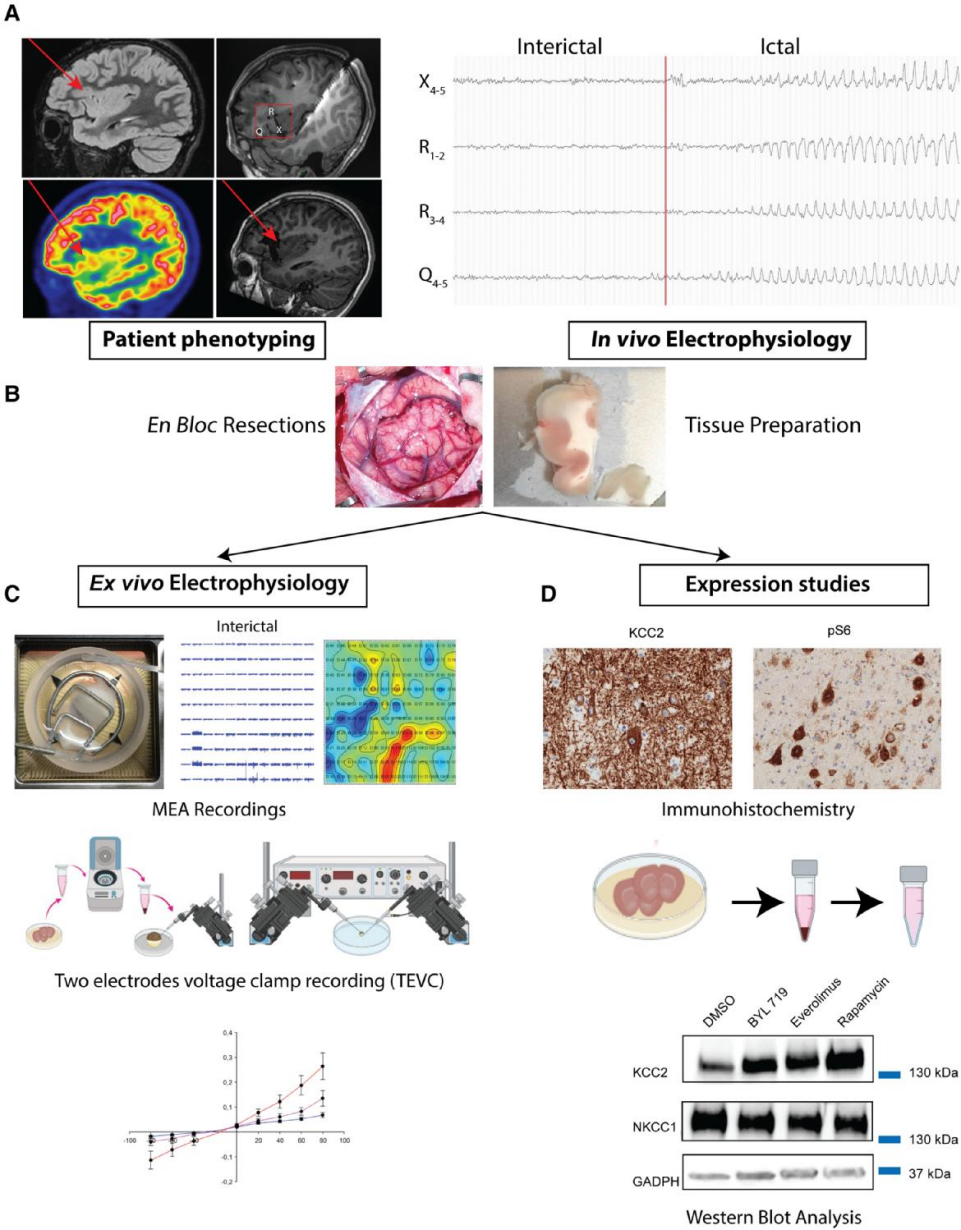
**Methodological illustration of the study.** (**A**) Presurgical evaluation. Imaging of a patient with focal cortical dysplasia (FCD) located in the vicinity of the circular sulcus of the right insular lobe. Red arrows indicate the FCD, with increased intensity on FLAIR sagittal MRI (*top left*) and ^18^F-FDG PET hypometabolism (*bottom left*). Intracranial EEG recordings were performed with stereo EEG (T_1_-weighted MRI, *top right*), allowing focal resection (postoperative T_1_-weighted MRI, *bottom right*). Epileptic interictal activity was restricted to the proximal plots of the electrodes Q-R-X and, typical of this patient, consisted of a synchronizing pattern localized in the abnormal sulcus and anterior short insular gyrus. (**B**) Cortical dysplasia is ablated with subpial ‘*en bloc*’ resection, transferred to the laboratory in oxygenated sucrose-based artificial CSF (aACSF), and the tissue is then cut into 400-µm-thick slices. (**C**) An electrophysiology study was performed with a 10 × 12 multi-electrode array that allows extensive mapping of spontaneous epileptic activity in aACSF or under pharmacological modulation in the bathing solution. Membrane preparation and micro-transplantation into *Xenopus* oocytes allows the study of GABA-induced currents and *E*_GABA_. (**D**) Expression studies allow analysis of expression of chloride cotransporters in basal conditions and after pharmacological modulation, with immunohistochemistry, immunofluorescence and western blot analysis.

Brain cortical samples were obtained from children undergoing epilepsy surgery between March 2021 and June 2023. A comprehensive presurgical evaluation was performed with EEG-video monitoring, 3 T MRI read by an experienced neuroradiologist, neuropsychological evaluation, genetic testing and, when required, ^18^FDG-PET or intracranial EEG recordings with depth electrodes (Fig. 1). The surgery was not modified for the study and consisted of ‘*en bloc* resections’ to avoid excessive manipulation of the tissue, as previously reported.^32^

We included patients operated for MTOR-related MCDs and a ‘pseudo-control’ group of epileptic children in whom cortical samples were taken around a brain lesion (peritumoural cortex, peri-stroke, Rasmussen). Histological examination confirmed the absence of tumour or mTOR-related histological abnormalities in the pseudo-controls.

### *In vitro* electrophysiology recordings and analysis

Brains samples were immediately placed in iced sucrose artificial CSF (aACSF) and transferred to the laboratory, where meninges and blood vessels were gently removed to allow slicing with a vibratome.^38^ The aACSF consisted of (in millimolar): 124 NaCl, 3 KCl, 26 NaHCO_3_, 1.6 CaCl_2_, 1.3 MgCl_2_ and 10 D-glucose, at pH 7.4. After storage in an interface chamber with continuous oxygenated aACSF perfusion at 37°C for 1 h, 400-μm-thick cortical slices were then recorded in a 12 × 10 multi-electrode array (MEA, MultiChannel Systems) bathed with oxygenated aACSF at 37°C to record spontaneous cortical activity, as previously described.^32,38,39^ Pharmacological studies on spontaneous interictal discharges (IIDs) were performed in modified aACSF containing *N*-ethylmalmeide (0.5 mM) and staurosporine (8 μM) to block WNK1/SPAK-OSR1 kinases, BYL719 (10 μM), everolimus (1 μM) and rapamycin (0.1–0.5 μM) to block the PI3K/AKT/MTOR pathway.

Local field potentials were then analysed (amplitude, width and frequency) with Clampﬁt software (pClamp Software; Molecular Devices), using visual analysis and the automated event detector toolbox via threshold detection to quantify IIDs on active electrodes in physiological and pharmacological conditions. We also performed time–frequency power spectrum analysis of the MEA signal. Raw data were preprocessed offline using software developed in-house and MATLAB code, as follows: (i) DC removal; (ii) low-pass filter (cut-off frequency: 200 Hz); (iii) signal downsampling (subsampling rate of 1:20, downsample from a sampling frequency of 10000 samples/s to 500 samples/s); and (iv) visual inspection for possible noisy electrode rejection. Time–frequency analysis was computed using fractional adaptive superlets (FASLT), a super-resolution method providing sharp representations across the whole frequency domain based on a set of wavelets.^40,41^ FieldTrip and in-house MATLAB code was used to illustrate epileptogenic activity power topographic distribution across the slice.^42^ For measuring the effect of drug application, power averages per electrode across the whole time–frequency spectrum were computed for the active electrodes (electrodes with the most pronounced epileptogenic activity) and represented as heat maps over the MEA array.

### Biochemistry studies

#### Expression analysis

Membranes were prepared as previously described.^43^ Briefly, the cortex sample was homogenized in a Polytron system in 10 volumes of an ice-cold buffer containing 50 mM Tris pH 7.4, 0.32 M sucrose, 5 mM EDTA and 1 mM EGTA supplemented with complete protease inhibitor (Roche). The homogenate was centrifuged at 800–1000*g* for 10 min (4°C). The supernatant was collected and centrifuged at 100 000*g* for 45 min (4°C). The pellet (P2) was osmotically shocked in 10 volumes of ice-cold water. The microsome suspension was centrifuged at 100 000*g* for 45 min (4°C). The remaining pellet (P3) was suspended in a buffer containing 10 mM HEPES (pH 7.4) and 10 mM KCl, then centrifuged again at 100 000*g* for 45 min (4°C). The last pellet (P4) was stored at −80°C until use. Protein concentrations were tested using a BCA Protein Assay Kit. Lysates (20–30 μg) were separated by 8% sodium dodecyl sulfate–polyacrylamide gel electrophoresis (SDS-PAGE) and transferred to a nitrocellulose membrane. The membranes were then immunoblotted in 5% (w/v) non-fat powdered milk in Tris-PBS with the indicated primary antibodies overnight at 4°C. The blots were then washed three times with Tris-PBS and incubated for 1 h at room temperature with secondary horseradish peroxidase-conjugated antibodies diluted 5000-fold. GAPDH was used as an internal control. After repeating the washing steps, the signal was detected using an enhanced chemiluminescence (ECL) reagent. Immunoblots were developed using ChemiDoc™ Imaging Systems (Bio-Rad, Feldkirchen). Densities of bands were measured with ImageJ.^44^

#### Co-immunoprecipitation assays

Co-immunoprecipitation was performed to detect the physical interaction between mTORC2 and WNK1/SPAK-OSR1 by using the immunoprecipitation kit dynabeads protein G. Briefly, samples were homogenized using a Polytron system in ice-cold buffer. The homogenate was first centrifuged at a low speed of 800–1000*g* for 10 min to remove all debris. The supernatant was then centrifuged at 14 500*g* for 30 min. Immunoprecipitation of the antigens was achieved by a 60 min rotating incubation at room temperature of the resulting supernatant with 2 µg of anti-WNK1 Ab or anti-mSIN1 Ab. IgG Ab was used as a negative control, followed by the addition of protein G-conjugated magnetic beads for 60 min. The magnetic beads were washed three times before elution in Laemmli sample buffer. Proteins were resolved by SDS-PAGE and transferred to nitrocellulose membranes for western blot analysis. Anti-mTOR Ab and anti-SPAK/OSR1 Ab diluted to 1:1000 were applied overnight at 4°C. The bound proteins were detected using goat anti-rabbit diluted to 1:5000 coupled to horseradish peroxidase.

For the phospho-antibody immunoprecipitation experiment, total KCC2 and total NKCC1 were immunoprecipitated from the indicated tissue extracts.^45^ This was incubated with 4 μg of the indicated KCC2, NKCC1 antibody or 4 µg mouse IgG, conjugated to 15 μl of protein G–sepharose. Incubation was overnight at 4°C with gentle agitation. The immunoprecipitates were then washed three times with lysis buffer and, finally, eluted with the sample buffer. KCC2pThr^906^ or NKCC1 pThr^203,207,212^ antibody was detected via western blot. The bound proteins were detected by using a rabbit anti-sheep IgG-coupled horseradish peroxidase.

#### KCC2 biotinylation

Slices were treated with the drugs for 30 min with 95% O_2_/5% CO_2_ aACSF. Surface proteins were labelled with 1.0 mg/ml sulfo-NHS-SS-biotin in ice-cold aACSF for 45 min at 4°C, with bubbling 95% O_2_/5% CO_2_. Following biotinylation, slices were washed three times in ice-cold aACSF, then lysed in lysis buffer. Cellular debris were removed by centrifugation at 18 000*g* for 20 min at 4°C, and protein concentrations were determined using the BCA protein assay. One hundred microlitres of streptavidin was added to 600 µg of protein lysate, followed by rotating incubation overnight at 4°C. Beads were pulled down after each wash by gentle centrifugation. The proteins were eluted with 2× SDS reducing buffer and heated at 85°C. Western blotting was performed using an anti-KCC2 antibody.

#### Histology

Part of the cortical samples used for electrophysiological experiments were subjected to histological examination by experienced neuropathologists to ensure that the cortices used in MEA recordings were pathological, and they were considered as a ‘mirror block’. Samples presenting abnormal cortical cytoarchitecture and dysmorphic neurons, with or without balloon cells, were considered representative of the epileptogenic lesion and included in further immunohistochemical study. Histological diagnosis was performed on 3-μm-thick slices stained with haematoxylin–phloxine–saffron (HPS), using the three criteria of abnormal cortical architecture, dysmorphic neurons and balloon cells, as defined by the 2022 ILAE consensus classification of focal cortical dysplasia.^46^ Unstained 3-μm-thick slices of formalin-fixed, paraffin-embedded tissues were submitted for immunostaining using an automated Stainer (Dako Omnis). The following primary antibodies were used: KCC2 (1:1000, rabbit), phosphoS6 (1:400). In three patients (*n* = 9 slices), half the cortical slices were treated with rapamycin before fixation. HPS and immunohistochemistry slides were digitized using an Aperio AT2 slide scanner (Leica). Digital slides were then viewed using the IDS7 application (Sectra, Norway). Cell count was performed manually by repurposing the IDS7 mitotic counting tool, after a 1 mm^2^ cortical region of interest was chosen as representative of the sample. The total number of neurons (normal and dysmorphic) per square millimetre was measured, as were the number of neurons with cytoplasmic or membranous KCC2 immunostaining. Comparison of total, cytoplasmic and membranous KCC2 was done: (i) between controls and mTOR-mutated tissue (including, FCD, TSC and HMG cases); and (ii) before and after treatment with rapamycin.

#### Immunofluorescence assay

Formalin-fixed, paraffin-embedded sections (5-μm thick) were deparaffinated in xylene, and antigen retrieval was performed using a pH 9.0 buffer (Akoya Biosciences). After permeabilization in PBS supplemented with 0.1% bovine serum albumin and 0.2% Triton-X and blocking in PBS supplemented with 3% bovine serum albumin for 1 h, the slides were incubated overnight at 4°C in blocking buffer containing the following primary antibodies: NeuN (1:150, Merck ABN90); pS6 (1:100, Cell Signaling Technology 2211); and KCC2 (1:50, DSHB N1/12). Secondary antibodies (1:400, Alexa Fluor 488 goat anti-mouse, Alexa Fluor 568 goat anti-rabbit and Alexa Fluor 647 goat anti-guinea pig) were incubated for 30 min at room temperature. DAPI (1:1000, Invitrogen D3571) was used as a nuclear stain, and the slides were mounted using Immu-Mount (Epredia 9990402). Images were captured at 63× with the Spinning Disk Zeiss system (Intelligent Imaging Innovations), an Examiner.Z1 upright stand (Carl Zeiss), a CSU-W1 head (Yokogawa) and an ORCA-Flash 4.0 camera (Hamamatsu). The same parameters were applied to all images during capturing and processing (ImageJ).

#### Molecular diagnosis

DNA extracted from formalin-fixed, paraffin-embedded or frozen cortical samples was analysed by targeted next-generation sequencing of 36 genes (Supplementary material, NGS Panel) mainly involved in the mTOR signalling pathway. Briefly, all coding exons of selected genes were screened by paired-end sequencing reactions of 150 bp reads on a MiSeq platform (Illumina) after capture-based target enrichment. The bioinformatic analysis included the trimming of raw next-generation sequencing reads (FASTQ), mapping, variant calling, variant annotation and filtering. Somatic variant curation was performed as recommended in cancer.^47^

#### Micro-transplantation of GABA receptors from MCD cortical samples to *Xenopus laevis*

Membranes from MCD cortical samples were prepared and injected into oocytes as previously reported.^48,49^ Briefly, 0.5 g of previously frozen tissue was homogenized in 50 mM Tris pH 7.4, 300 mM sucrose, 1 mM EGTA and 5 mM EDTA. The filtrate was centrifuged for 10 min at 1000*g* twice. The supernatant was then centrifuged for 1 h at 100 000*g*. The membranes were suspended in water and kept frozen at −80°C until used for an experiment.

*Xenopus laevis* oocytes were obtained from the IBENS (Paris, France) *Xenopus* facility (husbandry authorization #D75-05-31; project authorization Apafis #28867-2020121814485893) and from TEFOR (Paris Saclay, France). They were prepared as previously described.^50^ Briefly, small pieces of ovary were gently shaken at room temperature for 2–3 h in a Ca^2+^-free ND96 (96 mM NaCl, 2 mM KCl, 1 mM MgCl_2_ and 5 mM HEPES at pH 7.4) solution supplemented with collagenase 1A. After addition of CaCl_2_ (1.8 mM) and carefully washing off the enzyme, defolliculated and healthy-looking stage V–VI oocytes were selected and allowed to recover for a few hours (≤24 h) before injection.^51^ The next day, each membrane preparation was injected into oocytes at a protein concentration of 2–5 mg/ml (50 nl volume). Membranes were always microinjected into the animal pole of the *X. laevis* oocyte near the equatorial band.^48^ The injected oocytes were incubated at 17°C for 24–48 h in ND96 (pH 7.4) for electrophysiological studies. The incubation media were supplemented with penicillin/streptomycin (100 units, 100 µg/ml). Two-electrode voltage-clamp experiments were performed using a TEV-200A amplifier (Dagan), 1 or 2 days after micro-transplantation co-injection. Oocytes were subsequently placed in a microchamber and superfused with ND96 (pH 7.4) while being punctured with two low-resistance (0.5–2.0 MΩ), 3 M KCl-filled microelectrodes. Currents were recorded in response to a voltage protocol consisting of 20 mV steps from −80 to +80 mV during 800 ms. The reversal potential of the GABA receptor was determined using solutions containing ND96 alone, ND96 + 250 µM GABA or ND96 + 250 µM GABA + 0,5 µM rapamycin. Whole-cell currents were recorded at a holding potential of −50 mV. The value of *E*_Cl_ was determined by the current–voltage relationship while using a solution with low Cl^−^ concentration (10 mM NaCl). Chloride was replaced by gluconate, and calcium was increased to 3 mM (to compensate for Ca^2+^ chelation by gluconate salt).

Data were filtered at 500 Hz, digitized using a Digidata 1440A analog-to-digital converter and analysed using Axon pClamp 10 software (Molecular Devices).

### Statistics and reproducibility

Results are expressed as means ± standard error (SE) or ± standard deviation (SD). Statistical analysis was performed using Sigma Plot version 11.0. Western blot data were analysed for statistical significance using Student’s two-tailed *t*-test. Electrophysiological experiments were studied with the non-parametric Kruskal–Wallis test, and the Wilcoxon test was used for repeated measurement on a single sample. Values of *P* < 0.05 were considered significant.

For time–frequency analyses, normality assumption analyses (Shapiro–Wilk test) have been performed, followed by parametrical tests (ANOVA with *post hoc* Tukey’s HSD/Tukey–Kramer tests and *t*-tests with Bonferroni correction where required); *n* is the number of western blots, slices or oocytes, and *N* is number of patients or frogs.

## Results

### Cohort, histology and molecular diagnosis

Thirty-five children were included in this study (Supplementary Table 1), with 25 somatic mTOR-related malformations of cortical development and 10 pseudo-control cases. In the mTOR group, the male to female ratio was 1.7, with a mean age at surgery of 6.2 years (range 0.5–18.8 years, SD 5.5 years). Focal resection was performed in 15 cases, and lobar or multilobar/hemispheric disconnection was done in *n* = 3 and 7, respectively. The samples were taken mostly in the frontal lobe (*n* = 10) and central region (*n* = 6). Histology was as follows: FCD (type IIa *N* = 8; type IIb *N* = 6), HMG (*N* = 6) or TSC (*N* = 5). The ‘pseudo-control’ group consisted of 10 epileptic children (four male, mean age 8.2 years, SD 3.3 years). The samples originated mainly from the central region (in five cases of hemispherotomy), and diagnosis was as follows: peri lesional cortex (tumour *N* = 3, vascular malformation *N* = 2), Rasmussen’s encephalopathy (*N* = 3) and stroke (*N* = 2).

**Figure 2:**
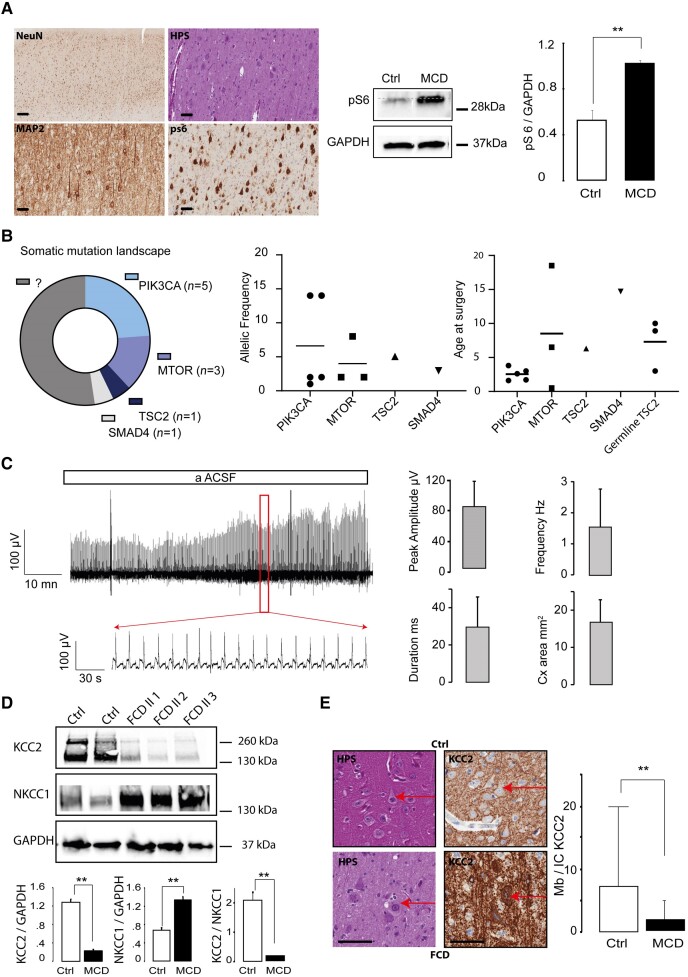
**Characteristics of focal cortical dysplasia cortical samples.** (**A**) Haematoxylin–phloxine–saffron (HPS) staining and immunostaining of a surgical specimen displaying a focal cortical dysplasia (FCD) type II with loss of cortical cytoarchitecture (anti-NeuN immunostaining; scale bar = 50 µm) and the presence of cytomegalic neurons with densified Nissl corpus (HPS and anti-MAP2 immunostaining; scale bars = 100 µm) and a strong pS6 cytoplasmic expression (anti-pS6; scale bar 100 µm). Scale bars in **A** = 500 µM. Dysmorphic neurons and balloon cells in FCD type IIb. Scale bars = 100 µM. Western blotting for pS6 in brain tissues from subjects with FCD type IIb and controls shows increased expression level of pS6 protein in brain tissues of FCD type II compared with controls. Densitometric analysis of the pS6 bands was quantified to GAPDH for each experimental condition. [*n* = 5 western blot, *N* = 3 malformations of cortical development (MCDs: two FCD and one TSC) and three controls (stroke/Rasmussen/peritumoural cortex)]. (**B**) Genomic analysis of the cohort shows mutations in the *PI3K/AKT/MTOR* with next-generation sequencing-identified somatic mutation in half of the cases with a low varient allele frequency (VAF). (**C**) MEA recordings of patient’s cortical slices *ex vivo* display spontaneous interictal discharges in physiological artificial CSF *in vitro*. The box plots illustrate the characteristics of the local field potentials in the MCD cohort. (**D**) KCC2 expression levels are reduced in all FCD II patients compared with controls. Brain slice lysates from control and MCD patients were probed for KCC2 and NKC1 levels. KCC2 and NKCC1 expressions were normalized to GAPDH. Nine independent western blot analyses are shown from *N* = 3 controls (stroke/Rasmussen/peritumoural cortex) and *N* = 6 MCD patients (two FCD, two TSC and two HMG). Reduced KCC2 expression levels are found in MCDs (0.199 ± 0.032 versus 1.281 ± 0.070; *P* ≤ 0.001). NKCC1 expression levels are increased in MCDs (1.253 ± 0.072 versus 0.674 ± 0.054; *P* ≤ 0.001). The KCC2/NKCC1 ratio is significantly reduced in FCDII compared with controls (1.253 ± 0.072 versus 0.674 ± 0.054; *P* ≤ 0001). (**E**) Immunohistochemistry analysis and cell counting show reduced membranous expression of KCC2 in the MTOR group. HPS and anti-KCC2 immunostaining from a pseudo-control (*top*, scale bar = 100 µm) and an MTOR patient (*bottom*, scale bar = 100 µm) illustrating membranous expression of KCC2 (Mb) in the pseudo-control group (Ctl) and intracytoplasmic accumulation of KCC2 (IC KCC2) in MTOR patients that was confirmed by cell counting.

A strong intracytoplasmic pS6 immunostaining was observed in cytomegalic cells (dysmorphic neurons and balloon cells) in all representative samples, confirming activation of the mTOR pathway (Fig. 2A), that was quantified further with western blot, because expression of pS6 was significantly higher (*n* = 5 western blot, *P* = 0.008) in MCDs cortex (*N* = 2: one FCDII and one TSC; 1.02 ± 0.02) than in pseudo-control cortex *N* = 3 (stroke/Rasmussen/peritumoral cortex; 0.52 ± 0.08).

Next-generation sequencing identified a single somatic mutation in 48% (10 of 21) patients with available tissue material. Fifty per cent (5 of 10) of the patients exhibited gain-of-function mutation in *PIK3CA* (two HMG, two tuber and one FCDIIA) except for one case of disruption, and 30% (3 of 10) had activating missense mutations in *MTOR* (one FCDIIa, one FCDIIb and one HMG). Next-generation sequencing also detected a somatic missense mutation in *TSC2* (*n* = 1) and a loss-of-function mutation in *SMAD4* (*n* = 1, FCDIIb) (Fig. 2B and Supplementary Table 1). Mutational variant allelic frequency ranged from 1% to 14% (median: 2%). Interestingly, all *PIK3CA* mutations were found in patients <4 years of age. In addition, germline mutations in *TSC2* were observed in all patients with TSC (*N* = 5), including one case with additional germline mutation in *PKD1* (Fig. 2B).

### MCD cortical slices display spontaneous epileptic events *in vitro* and altered CCC expression

Fifty-seven slices from 17 patients (six FCDIIa, four FCDIIb, four TSC and three HMG) were recorded on MEAs (median = three slices per patient). Spontaneous IIDs were recorded in 37 slices (65%) in physiological aACSF, with a mean frequency of 1.56 ± 1.19 Hz, a mean amplitude of 80.12 ± 32.52 µV and a mean duration of 29.65 ± 16.02 ms (Fig. 2C). The mean cortical area displaying IIDs was 16.85 ± 5.99 mm^2^, corresponding to 9.36% ± 3.33% of the cortex explored. These data are consistent with our previous reports.^32^

Western blot analysis showed an inversion of the KCC2/NKCC1 ratio in MCD tissue in comparison to control tissue (*n* = 9 western blots; *N* = 6 MCDs: two FCD, two TSC and two HMG; *N* = 3 controls: stroke/Rasmussen/peritumoural cortex). Mean expression of NKCC1 was 1.253 ± 0.072 in MCDs versus 0.674 ± 0.054 for controls (*P* < 0001), and values for KCC2 were 0.199 ± 0.032 in MCD versus 1.281 ± 0.070 for controls (*P* < 0.001). The KCC2/NKCC1 ratio was 1.253 ± 0.072 in MCD versus 0.674 ± 0.054 in controls (*P* < 0.001) (Fig. 2D).

Immunohistochemistry displayed an intracytoplasmic localization of KCC2 in dysmorphic neurons compared with the controls (Fig. 2E): membranous expression of KCC2 was reduced in the MTOR group in comparison to the control group, with a membrane/intracellular ratio of 2.01 ± 3.03 and 6.5 ± 14.6, respectively (*P* = 0.0001), with *n* = 9 slices and *N* = 3 patients.

### Chloride cotransporter expression is regulated by WNK1/SPAK-OSR1 kinases

**Figure 3:**
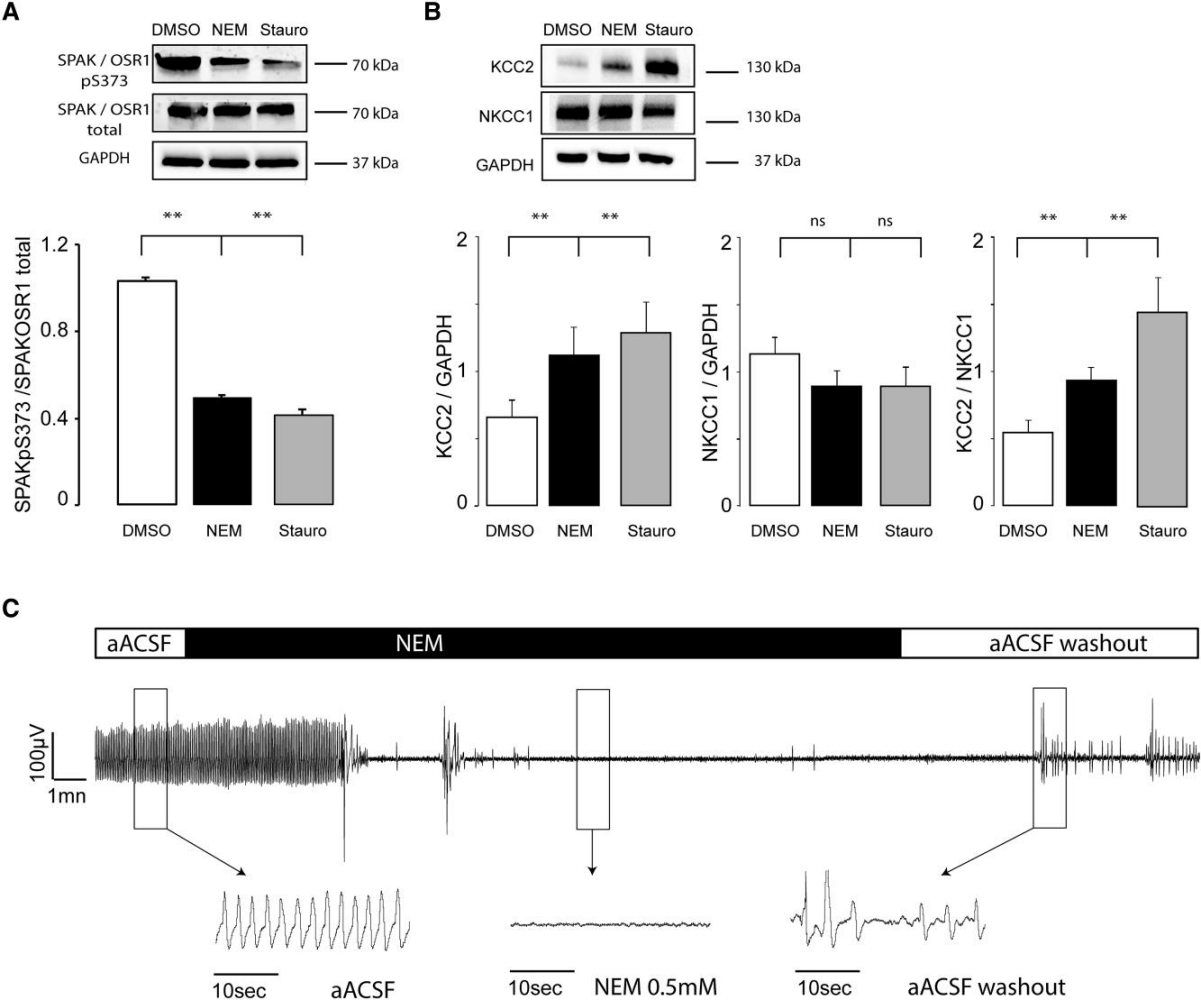
**WNK1/SPAK-OSR1 regulate chloride cotransporter expression in focal cortical dysplasia.** (**A**) *N*-ethylmaleimide (NEM) and staurosporine impair WNK1/SPAK-OSR1 phosphorylation. *Top*: Representative western blots and quantification of SPAK-OSR1pS^373^ expression levels of brain slice lysates in the presence of DMSO, NEM and staurosporine. *Bottom*: SPAK-OSR1pS^373^ expression was normalized to total SPAK-OSR1 (*n* = 4 and *N* = 3 patients with malformations of cortical development: two FCD and one HMG). SPAK-OSR1pS^373^/total SPAK-OSR1 ratio was lower (*P* < 0.001) in the presence of NEM (0.494 ± 0.025) and staurosporine (0.415 ± 0.025) than in DMSO (1.036 ± 0.016). (**B**) NEM and staurosporine treatment increases KCC2 expression and restores KCC2/NKCC1 ratio. *Top*: Representative western blot from brain slice lysates from treated with DMSO, NEM and staurosporine were probed for KCC2 and NKC1 levels. *Bottom*: NEM and staurosporine restore KCC2/NKCC1 ratio. KCC2 and NKCC1 expressions were normalized to GAPDH [*n* = 4 and *N* = 4 patients (two FCD, one HMG and one TSC)]. Error bars represent SE. Band intensities were quantified using ImageJ software. (**C**) Inhibition of WNK1/SPAK-OSR1 by NEM decreases epileptic activities *in vitro*. Representative recordings in a single electrode of spontaneous interictal activity recorded in a cortical slice from an FCD patient *in vitro* before (aACSF), during 0.5 mM NEM treatment and after washout with aACSF. NEM inhibited spontaneous seizures in human brain slices with malformations of cortical development.

To study the regulation of the CCCs NKCC1/KCC2 by the WNK1/SPAK-OSR1 kinases, we used *N*-ethylmaleimide (NEM) and staurosporine, which dephosphorylate the T loop and S loop phosphorylation sites Thr^233^ and Ser^373^ of SPAK, respectively (Fig. 3).^52^

Initially, we confirmed that treatment with NEM and staurosporine reduced the overall level of phosphorylated SPAK/OSR1 (Fig. 3A), because the SPAK-OSR1pS^373^/total SPAK-OSR1 ratio was lower in NEM (0.494 ± 0.025) and staurosporine (0.415 ± 0.025) conditions than in control [dimethyl sulfoxide (DMSO)] [1.036 ± 0.016; *P* < 0.001, *n* = 4 western blots and *N* = 3 MCD patients (two FCD and one HMG)]. Next we quantified KCC2 and NKCC1 expression in control conditions and in the presence of NEM or staurosporine and found a slight, yet non-significant decrease in NKCC1 (0.892 ± 0.114 versus 1.132 ± 0.125; *P* = 0.188 with NEM and 0.895 ± 0.139 versus 1.132 ± 0.125; *P* = 0236 with staurosporine) and a significant increase in KCC2 expression (1.118 ± 0.208 versus 0.661 ± 0.126; *P* = 0.024 with NEM and 1.284 ± 0.231 versus 0.661 ± 0.126; *P* = 0014 with staurosporine), resulting in the restoration of the KCC2/NKCC1 ratio (0.971 ± 0.077 versus 0.547 ± 0.092; *P* = 0005 under NEM and 1.447 ± 0.252 versus 0.547 ± 0.092; *P* = 0.007 under staurosporine) *n* = 6 western blot and *N* = 4 MCD cortices (Fig. 3B).

Interestingly, restoration of the expression of CCCs resulted in suppression of the interictal epileptic activity (Fig. 3C) on MEA recordings (*n* = 5). Indeed, IIDs were stopped in three slices after a mean delay of 30 min of bathing in aACSF with NEM (0.5 mM). In the remaining two cases, the mean IID frequency and amplitude reduced from 1.932 ± 0.594 to 1.669 ± 0.726 Hz and from 55.228 ± 27.159 to 13.106 ± 9.699 µV, respectively.

### MTOR interacts with WNK1/SPAK-OSR1 kinases

**Figure 4:**
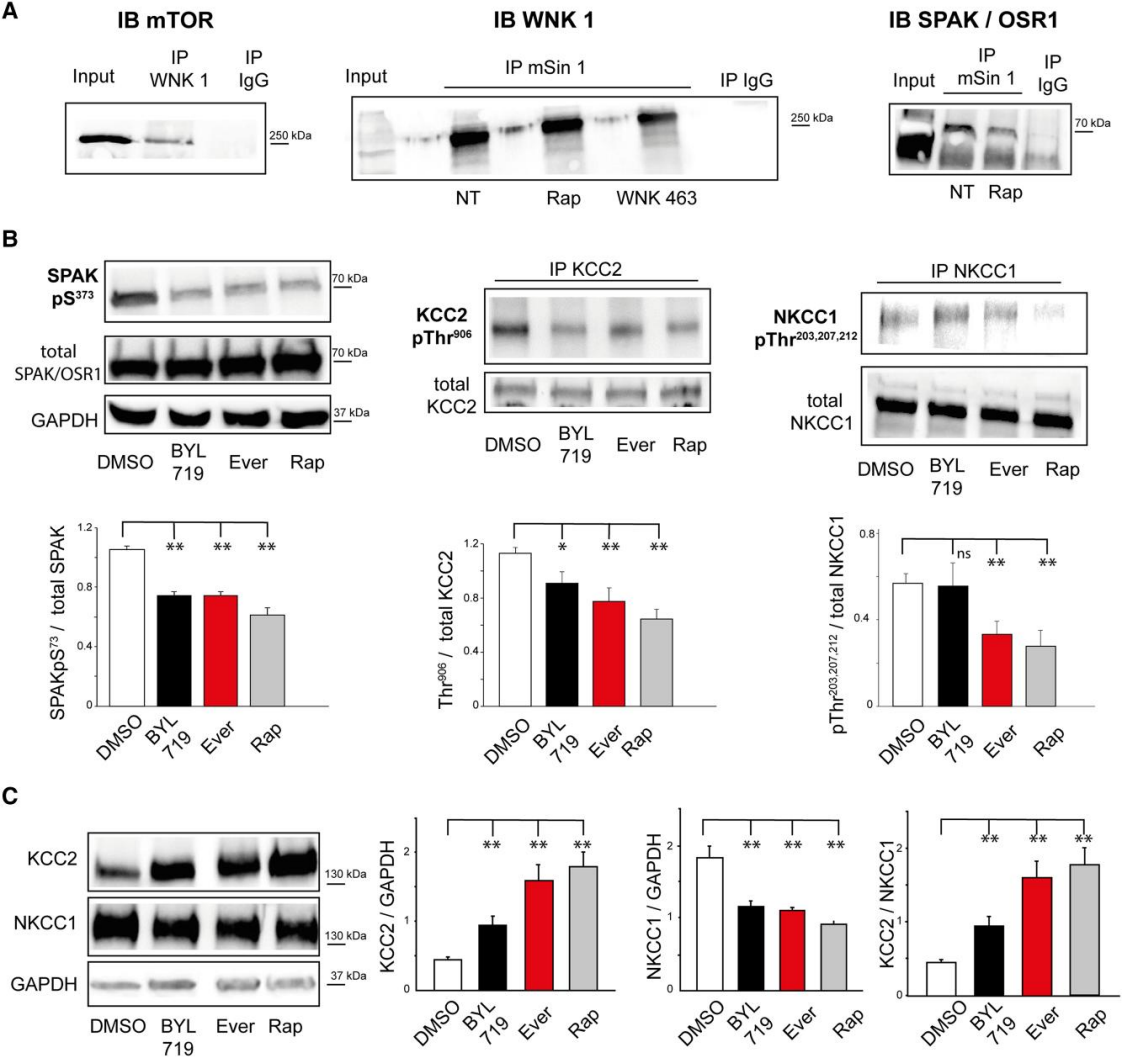
**Phosphorylation cascades underlying mTOR and CCC interactions.** (**A**) MTOR and WNK1/SPAK-OSR1 have a direct physical interaction that is inhibited by rapamycin. *Left*: WNK1 protein was immunoprecipitated (IP) with an anti-WNK1 antibody, the immunoprecipitate was probed further with anti-mTOR antibody to detect mTOR on western blot. *Middle*: Brain slices were pretreated with DMSO, rapamycin or WNK463 inhibitor for 30 min before lysis. The lysate was immunoprecipitated with an antibody directed against mSIN1 and analysed by western blot with anti-WNK1. *Right*: Brain slices were pretreated with DMSO or rapamycin for 30 min before lysis. The protein interaction of SPAK/OSR and mSIN1 was assessed by co-immunoprecipitation. (**B**) Inhibition of mTOR dephosphorylates WNK1/SPAK-OSR1 and chloride cotransporters*. Left*: Immunoblots (IB) for evaluating changes in SPAK-OSR1pS^373^ expression. GAPDH was used as a loading control. [*n* = 4 and *N* = 4 patients with malformations of cortical development (two FCD, one HMG and one TSC)]. SPAK-OSR1pS^373^/total SPAK-OSR1 ratio was lower in BYL719, everolimus and rapamycin conditions than in DMSO. Error bars represent the SE. *Middle and right*: Quantitative analyses of KCC2 pThr^906^ and NKCC1pTh^203,207,212^ upon BYL719, everolimus and rapamycin treatment in neurons in brain slices [*n* = 6–4 western blot respectively and four patients (two FCD, one HMG and one TSC)]. Slices were treated with DMSO (control),10 μM BYL719, 1 µM everolimus or 0.5 µM rapamycin, respectively, for 30 min. Immunoprecipitated KCC2 and NKCC1 were probed for KCC2pThr^906^ and NKCC1pTh^203,207,212^. Densitometric analysis of the KCC2pThr^906^ and NKCC1pTh^203,207,212^ bands was performed after normalization to total KCC2 and total NKCC1 for each experimental condition. (**C**) mTOR inhibition restores the KCC2/NKCC1 ratio. Representative western blot of proteins of slice lysates (*left*) probed with KCC2, KCC1 and GAPDH antibodies. Densitometric analyses of the KCC2 and NKCC1 bands were quantified to GAPDH for each experimental condition [*n* = 6 western blots and *N* = 5 patients (two FCD, two HMH and one TSC)]. Bar graph shows relative levels of KCC2/GAPDH, NKCC1/GAPDH and KCC2/NKCC1 ratio.

To analyse putative physical interactions between mTOR protein and WNK1/SPAK-OSR1, we performed co-immunoprecipitation experiments in the MCD cortical lysates using antibodies directed against mTOR and the mSIN1 subunit of mTORC2. The precipitate was analysed further with antibodies against WNK1 and SPAK/OSR1. Our result revealed that endogenous mTOR and mSIN1 interact with WNK1 and SPAK/OSR1 (Fig. 4A). The binding interaction between SPAK/OSR1 and mSIN1 appears to be dependent on the activation status of the two kinases, because application of the WNK inhibitor WNK463 or rapamycin caused a reduced immunoprecipitation of WNK1 with mSIN1 (*n* = 2 and *N* = 2 MCD: one FCD and one HMG for each condition). In control conditions where normal purified IgG was used for immunoprecipitation, we saw no detectable endogenous co-immunoprecipitation of the two proteins. In support of a functional interaction between mTOR and SPAK/OSR1, we found a decrease in the phosphorylation level of SPAK-OSR1pS^373^ when we treated the cortical samples with inhibitors of the PI3K/AKT/MTOR pathway BYL719 (0.74 ± 0.02), everolimus (0.75 ± 0.02) or rapamycin (0.60 ± 0.04) versus 1.05 ± 0.02 in DMSO (*n* = 4 and *N* = 4 patients; *P* < 0.001) (Fig. 4B).

### MTOR inhibition restores KCC2 membrane insertion

To analyse the functional interactions between the MTOR protein network and CCCs, we studied the phosphorylation levels of NKCC1 and KCC2 while inhibiting the mTOR pathway. To do this, we performed immunoprecipitation after treatment with BYL719, everolimus and rapamycin using specific antibodies for KCC2pThr^906^ and NKCC1 pThr^203,207,212^ to precipitate KCC2 and NKCC1 (Fig. 4B). The ratio of KCC2pThr^906^ to total KCC2 (*n* = 6 western blot and *N* = 4 MCD: two FCD, one TSC and one HMG) was significantly decreased in the lysate following treatment with BYL719 (1.12 ± 0.04 versus 0.90 ± 0.08, *P* = 0.026), everolimus (1.12 ± 0.04 versus 0.77 ± 0.09 versus, *P* < 0.001) and rapamycin (1.12 ± 0.04 versus 0.64 ± 0.06, *P* < 0.001) respectively compared with untreated (Fig. 4B). Both everolimus (0.33 ± 0.06 versus 0.57 ± 0.04, *P* = 0.002) and rapamycin (0.28 ± 0.07 versus 0.57 ± 0.04, *P* = 0.002) reduced the phosphorylation ratio of NKCC1 pThr^203,207,212^ compared with total NKCC1, whereas BYL719 did not modify the dephosphorylation of NKCC1 pThr^203,207,212^ (0.55 ± 0.11 versus 0.57 ± 0.04) relative to total NKCC1 (*P* = 1) (Fig. 4B) with *n* = 4 western blots and *N* = 4 MCD patients (two FCD, one TSC and one HMG).

**Figure 5:**
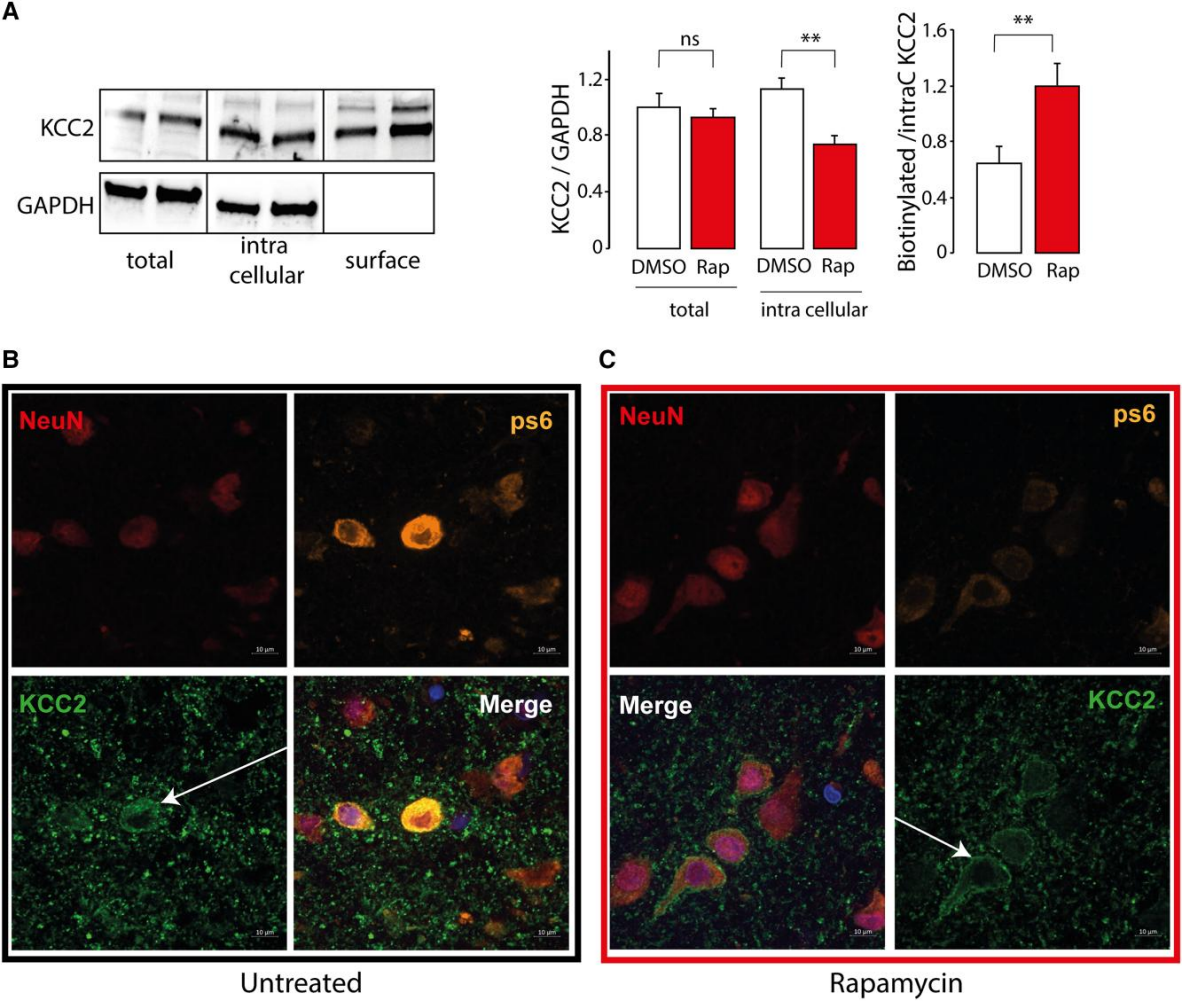
**mTOR inhibition restores KCC2 membrane insertion.** (**A**) Quantification of membranous KCC2. Brain slices were pretreated with vehicle (DMSO) or rapamycin for 30 min before lysis. Slices were then labelled with biotinylation reagent. Biotinylated surface proteins were then separated by streptavidin resin. *Left*: Representative western blot of total, cytosolic and biotinylated surface proteins of slice lysates probed with GAPDH and KCC2 antibodies. *Right*: Bar graph shows relative total and cytosolic levels of KCC2/GAPDH proteins (*N* = 2 patients with malformations of cortical development and *n* = 6 western blots). Densitometric analysis of the biotinylated KCC2 band was performed after normalization for the intracellular amount of KCC2 for each experimental condition. (**B** and **C**) Subcellular KCC2 repartition immunofluorescence imaging of FCD in basal conditions (**B**, *left*, black rectangle) and after mTOR inhibition (**C**, *right*, red rectangle) in two different slices. Neuronal (NeuN) KCC2 in pS6^+^ cells is located in the cytoplasm in FCD, and on the membrane after rapamycin treatment.

Given that phosphorylation of KCC2 is related to its degree of internalization, we then performed a biotinylation assay to quantify membranous, total and cytosolic KCC2 expression (Fig. 5). Quantitative analysis (Fig. 5A) revealed no significant difference in total KCC2 expression between non-treated and rapamycin-treated lysates (KCC2/GADPH ratio: 0.99 ± 0.1 versus 0.92 ± 0.06; *P* = 0.56). In contrast, rapamycin decreased the intracellular fraction of KCC2 in comparison to DMSO (0.73 ± 0.05 versus 1.12 ± 0.08, *P* = 0.002). along with an increase in the membranous expression of KCC2 (KCC2/GAPDH ratio: 1.1861 ± 0.15 versus 0.63 ± 0.12, *P* = 0.002). This suggests that rapamycin rescues the expression of KCC2 at the membrane, through dephosphorylation of KCC2 Thr^906^. Immunofluorescence analysis in MCD cortical samples treated with rapamycin confirmed its dramatic effect on the membrane inclusion of KCC2. Non-treated MCD neurons are characterized by a uniformly distributed cytoplasmic signal of the anti-KCC2 antibody (Fig. 5B), and 30 min of rapamycin treatment was sufficient to drive the bulk of KCC2 to the membrane and to clear the cytoplasmic signal (Fig. 5C).

### MTOR inhibition restores the equilibrium potential for GABA and has an anti-epileptic effect

**Figure 6:**
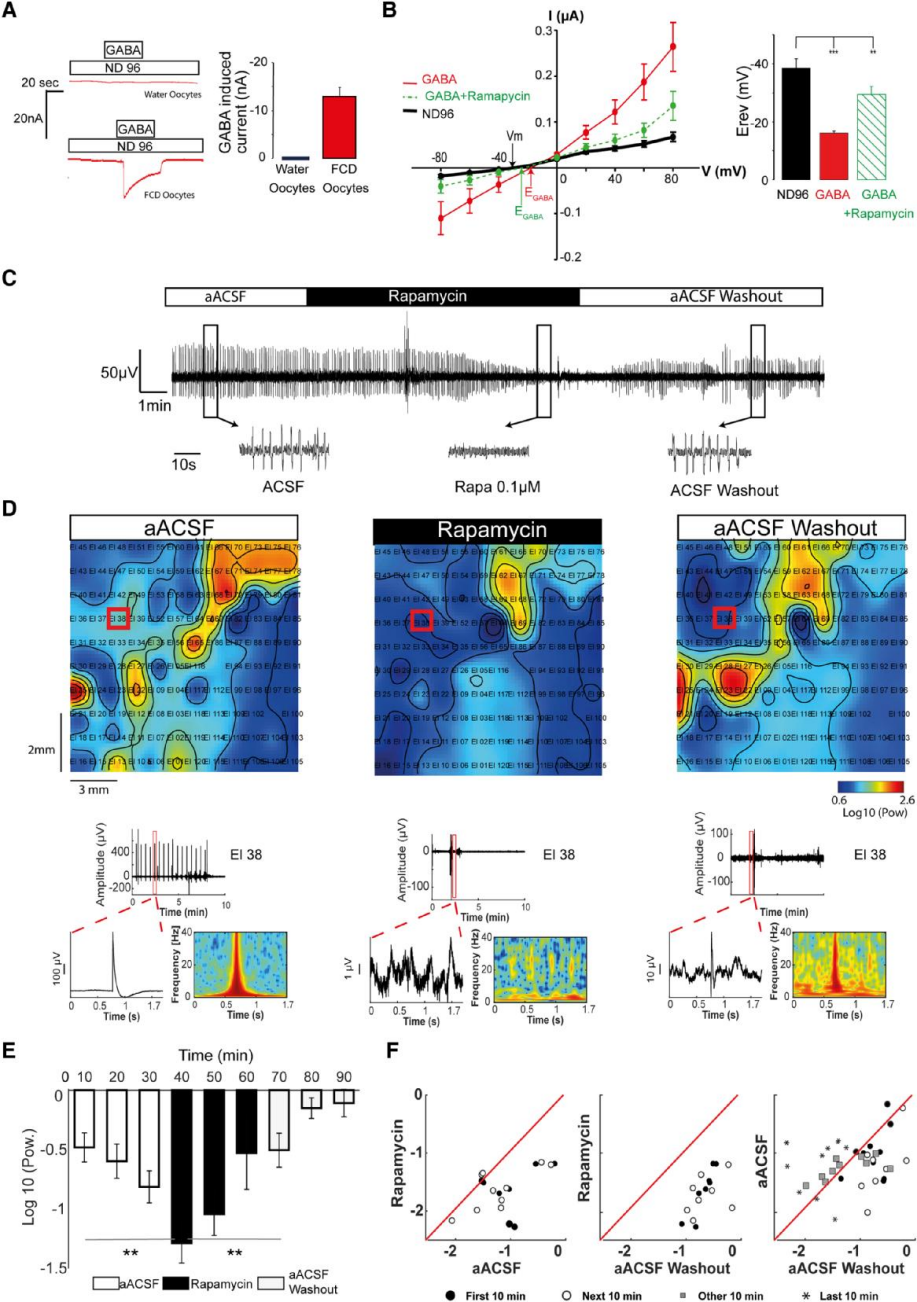
**mTOR inhibition restores the equilibrium potential for GABA and blocks spontaneous interictal discharges *ex vivo*.** (**A**) GABA-induced current. *Left*: GABA-induced current (250 µM of GABA added in ND96) was measured at holding potential of −50 mV in oocytes incorporating membrane from a focal cortical dysplasia (FCD) patient [*n* = 15 oocytes, *N* = 3 frogs and three patients (two FCD and one HMG)]. Oocytes injected with water were used as control oocytes. Original traces show the current induced by the addition of 250 µM GABA in the superfusing medium. *Right*: Bar graph of the experiments display the effect of 250 µM in both types of oocytes. (**B**) Equilibrium potential for GABA (*E*_GABA_) depolarization. *Left*: Current–voltage relationship from oocytes injected with membranes of human brain FCD under ND96 (black), GABA (red) and GABA + rapamycin (green). The points represent means ± SE of peak GABA currents. *E*_GABA_ was determined as the intercept of the current–voltage curve with the *x*-axis: *E*_GABA_ 16.28 ± 0.59 mV; *E*_GABA_ shifts to −29.5 ± 10.24 mV after rapamycin treatment [*P* = 0.007, paired *t*-test; *n* = 14 oocytes, *N* = 3 frogs and *N* = 3 patients (two FCD and one HMG)]. *Right*: Bar graph illustrating the mean ± SE of *E*_rev_ for oocytes under ND96 (black), GABA (red) and GABA + rapamycin (green). Note that the mean *E*_GABA_ was significantly more positive in FCD tissues before rapamycin treatment. Rapamycin application shifts *E*_GABA_ to more negative potential. Data represented are pooled from three separate experiments, with four to five oocytes per experiment (*N* = 3 frogs and 3 malformations of cortical development, *n* = 14 oocytes). (**C**) Rapamycin inhibits spontaneous interictal discharges in FCD slices *ex vivo*. Representative local field potential recordings from a single channel during MEA recordings before pharmacological modulation in aACSF, under rapmycin (0.5 µM) treatment and after washout with aACSF. (**D**) Heat map examples for the 2–40 Hz frequency range, for aACSF, rapamycin and washed. The heat maps illustrate the frequency topographic distribution of the data (time window = 10 min), according to the interictal discharges on the 120 contacts of the MEA grid. The heat maps use one average value [log_10_(Pow)] for each electrode, using interpolation to calculate and plot values between the electrodes. The red square indicates an active electrode that is illustrated further below. In aACSF, a power distribution pattern, potentially associated with spreading, can be observed. This pattern is suppressed in rapamycin but reactivated after aACSF washout. Time-resolved superlet spectra for the active electrode reveal rapamycin suppression and washed power reactivation. (**E**) Bar plots for all the active electrodes spectral power averages (log_10_), for aACSF, rapamycin and washout. The bars represent continuous series of averages over a 10 min interval. Error bars = SEM. **P < 0.01.

To test whether the effect of rapamycin on the KCC2 reinsertion into the membrane has a direct effect on the regulation of intracellular chloride content, assessed by the equilibrium potential for GABA (*E*_GABA_), we used the oocyte heterologous testing system. Previous research has shown that membrane extracts from human epileptic brains are associated with a depolarizing shift in the GABA reversal potential when injected into oocytes and linked to a different ratio of membranous NKCC1/KCC2 in epileptic versus control membrane preparations.^51,53^ We therefore investigated whether rapamycin treatment could influence GABA_A_-induced currents of MCD membrane-injected oocytes. Oocytes injected with MCD membranes, but not control water-injected oocytes, responded to GABA exposure with an inward current (−12.93 ± 1.91 nA, *n* = 15 oocytes *N* = 3 *Xenopus* and *N* = 3 patients: two FCD and one HMG) (Fig. 6A). The reversal potential calculated from the current–voltage relationship was −16.28 ± 0.59 mV, similar to the previously reported value (−18.5 ± 0.2 mV).^52^ The value of *E*_Cl_ in the MCD oocytes was determined by current–voltage curves while using a low-chloride solution (10 mM NaCl). Mean *E*_Cl_ was −24.44 ± 1.68 mV (*n* = 10 MCD oocytes), which is comparable to the *E*_Cl_ in native non-injected *Xenopus* oocytes.^53^ Treatment of the injected oocytes with rapamycin (0.5 µM) was able to shift *E*_GABA_ to a significantly more negative potential (−29.5 ± 10.24 mV, *n* = 14 oocytes; *N* = 3 frogs and *N* = 3 MCD patients), in line with the predicted effect of rapamycin on KCC2 membrane reinsertion (Fig. 6B). In our conditions, *E*_GABA_ in MCD injected oocytes is close to the *E*_Cl_ in *Xenopus* oocytes. This suggests that the shift of *E*_GABA_ towards positive potentials is not the consequence of a change in chloride transmembrane gradient of the whole oocyte but is related to functional imbalance of NKCC1 and KCC2 in MCDs.

To determine the functional consequence of restored KCC2 expression by rapamycin treatment on the epileptic activity, we performed MEA recordings on the treated human MCD cortical slices. We found that bathing in the aACSF solution with rapamycin blocked interictal epileptic activity in seven slices after a mean delay of 15 min (Fig. 6C). Removal of the rapamycin restored interictal activity in these slices, thus confirming that MTOR inhibition with rapamycin has an anti-epileptic effect on human cortical MCD slices *ex vivo*.

The functional effect of rapamycin was also assessed with spectral analyses of the MEA signal. Heat maps of average power for individual slices revealed a suppression effect of rapamycin on the power spectrum in both the low-frequency (0.6–2 Hz; Supplementary Fig. 1) and high-frequency (2–40 Hz; Fig. 6D–F) ranges compared with aACSF. We observed a pattern of rhythmic bursts, potentially involved in epileptic activity spreading, which was significantly reduced by rapamycin treatment (power mean = 1.87, SD = 0.22 versus aACSF, mean = 2.15, SD = 0.19, *t*(2.66) = 14, *P* = 0.01). Removal of rapamycin reverted this effect (aACSF versus washed, *P* = 0.96), in line with the observations on ictal activity.

## Discussion

Here, using electrophysiology, biochemistry and pharmacological modulation *ex vivo* in human FCD type II, HMG and TSC, we show physical interactions between mTOR and WNK1/SPAK-OSR1 kinases leading to the phosphorylation of the chloride cotransporters NKCC1/KCC2, resulting in a depolarizing shift of *E*_Cl_ and, ultimately, hyperexcitability and epilepsy.

Chloride cotransporter expression is a key regulator of neuronal excitability through cerebral development; high levels of NKCC1 during embryonic life lead to increased neuronal chloride and GABA depolarization, while progressive expression of KCC2 after birth in humans shifts *E*_Cl_ negatively, and GABA becomes inhibitory in a caudal–rostral progression.^54^ Interestingly, embryonic-like expression of chloride cotransporters was reported in human MCDs. Increased expression of NKCC1 was noted on both astrocytes and neurons from MCD,^27^ whereas a global neuronal decreased expression of KCC2 was displayed along with abnormalities of its cell localization, with rare membranous and important cytosolic immunoreactivity.^28,30^ However, if these results suggest either a default in membrane addressing or an excess in endocytosis, chloride cotransporter deregulation in MCDs has not yet been deciphered. CCCs are involved in cell volume and osmolarity under the post-translational control of the WNK1/SPAK-OSR1 kinases that sense these cell modifications. WNKs stimulate SPAK-OSR1, which directly phosphorylates CCCs, stimulating the NKCCs and inhibiting the KCCs, resulting in chloride influx.^33^ This pathway is deregulated in several neurological disorders (autism, schizophrenia and hydrocephalus),^55^ and particularly in neuropathic pain, in which the WNK1-dependent high chloride level in the dorsal horn results in a default of GABA inhibition.^34^ Here, we show, for the first time in human MCDs, such a deregulation of NKCC1 and KCC2 expression levels, because specific inhibition^52^ of WNK1/SPAK-OSR1 with NEM and staurosporine restores a high KCC2/NKCC1 ratio and blocks epileptic activities.

In the context of mTORpathies, we then investigated a possible link between mTOR and WNK1/SPAK-OSR1 at the post-translational level. Indeed, AKT directly phosphorylates WNK1, indirectly phosphorylates WNK3–4,^35^ and a physical interaction between the mSIN1 subunit of mTORC2 and SPAK-OSR1 was found in HeLa cells, and between mSIN1 and WNK1 in the kidney.^36,56^ Here, we confirm, in human paediatric MCDs through co-immunoprecipitation assays, that endogenous mTOR and mSIN1 interact with WNK1, and that mSIN1 interacts with SPAK-OSR1. The presence of these physical interactions led us to explore whether hyperactivation of the mTOR pathway was involved in changes in the phosphorylation level of KCC2 and NKCC1. While blocking this signalling at different levels with BYL719, everolimus and rapamycin, we could show decreased phosphorylation of Ser^373^ in SPAK-OSR and Thr^906^ in KCC2. Both everolimus and rapamycin reduced phosphorylation of Thr^203,207,212^ of NKCC1 in MCD slices, while BYL719 did not significantly affect this level, perhaps because it was acting upstream of the mutated MTOR. We also demonstrated that kinase-dependent phosphorylation interactions between mTOR/WNK1/SPAK-OSR1/NKCC1/KCC2 led to a modification of the membranous KCC2/NKCC1 ratio and that decreased phosphorylation of mSIN1 by rapamycin resulted in a decrease in mSIN1/WNK1 and mSIN1/SPAK-OSR1 interaction. We showed with biotinylation approaches that the phosphorylation level of C-terminal Thr^906^ is a key determinant of KCC2 subcellular localization at the membrane. Activation of the mTOR pathway therefore contributes to KCC2 membranous declustering through phosphorylation cascades.

Given that KCC2 downregulation leads to a depolarizing shift of *E*_Cl_ and GABA depolarization in the subiculum in hippocampal sclerosis,^57^ we eventually studied chloride conductance of MCD membranes using *Xenopus* oocytes. Two-electrode voltage clamp showed that GABA exposure led to depolarization, confirming previously published data of the paradoxically depolarizing neuronal effect of GABA in MCDs at the network level on human cortical slices^22,32^ and at the single-cell level.^23,58^ Inhibition of the MTOR pathway with rapamycin restored *E*_GABA_ on *Xenopus* oocytes by inhibiting chloride accumulation and suppressed epileptiform activity on the patient’s MCD slices *ex vivo*, in line with anti-epileptic effects reported in randomized controlled trials of children with TSC treated with everolimus.^59,60^ Furthermore, a time–frequency analysis revealed a pattern of rhythmic oscillations, potentially involved in spreading epileptic activity, reduced by rapamycin application, suggesting an active effect of the drug in controlling epileptic propagation.

## Conclusion

Altogether, our data highlight one of the possible mechanisms of neuronal hyperexcitability in MCDs, in which mTOR hyperactivation leads to GABA depolarization. This pathway can be targeted at multiple levels, paving the way for adjunctive specific anti-epileptic drugs in FCD.

## Supplementary Material

awae262_Supplementary_Data

## Data Availability

Data supporting the findings of this study are available on request from the study authors.
