# Supplementary material for: Chloride deregulation and GABA depolarization in MTOR-related malformations of cortical development
Source: Brain. 2024 Aug 6;148(2):549–63. doi: 10.1093/brain/awae262 (PMC11788215; doi:10.1093/brain/awae262)
Supplement: awae262_Supplementary_Data [file awae262_supplementary_data.pdf]

Supplementary Data

Suppl Table 1: Antibodies and Reagent

REAGENT	SOURCE	IDENTIFIER
Antibodies		
Sheep KCC2A pThr906	University of Dundee	S959C
Rabbit KCC2	Proteintech	19565-1-AP
Mouse KCC2	DSHB	N1/12
sheep SPAK/OSR1 pSer373/Ser325	University of Dundee	S204C
Sheep NKCC1 pThr203+pThr207+pThr212	University of Dundee	S063D
Rabbit NKCC1	Proteintech	13884-1-AP
Sheep WNK1	University of Dundee	S062B
Rabbit mTOR	Cell Signaling Technology	2983
Rabbit phospho RPS6	Cell Signaling Technology	2211
Rabbit mSIN1	Sigma Aldrich	072276-1
Mouse NeuN	Merck	ABN90
Chiken MAP2	Merck	AB15452
Rabbit GAPDH	Proteintech	10494-1-AP
Mouse IgG	Millipore	12-371
Mouse Alexafluor 488	Thermo Scientific	A11001
Rabbit Alexafluor 568	Thermo Scientific	A11008
Goat Alexafluor 647	Thermo Scientific	A21450
Chemicals		
Rapamycin	life technologies	PHZ1235
Alpelisib BYL719	MedChemTronica	HY-1524
WNK463	Sigma	SML2809
Everolimus	Sigma	SML2282
Staurosporine	Merck	19-123
N-Ethylmaleimide	Merck	E3876
InvitrogenDynabeads Protein G Immunoprecipitation Kit	Thermo Fisher Scientific.	10278723
EZ-LinkTM Sulfo-NHS-SS-biotin	Thermo Fisher Scientific.	21441
BCA Protein Assay Kit	Thermo Fisher Scientific.	23225
Streptavidin agarose resin	Thermo Fisher Scientific.	20349
Gamma aminobutyric acid	Sigma.	A2129
collagenase 1A	Sigma.	C9891
Software and algorithms		
Image	thermo Fisher Scientific.	https://imagej.nih.gov/ij/
Sigma plot	Systat software Inc	https://sigmaplot.software.informer.com
Clampex 10	Molecular devices	https://www.moleculardevices.com
McRack	Multi Channel Systems	https://www.multichannelsystems.com
Multi-electrode array (MEA)	Multi Channel Systems	https://www.multichannelsystems.com

NGS Panel

DNA extracted from FFPE or frozen cortical samples was analyzed by targeted Next-Generation Sequencing (NGS) of 36 genes mainly involved in the mTOR signaling pathway (*ACRVL1, AKT1, AKT2, AKT3, ARID1A, BMP9, BMPER, BRAF, CCM2, CCM3, ENG, EPHB4, GJA4, GNA11, GNA14, GNAQ, HRAS, IDH1, IDH2, KRAS, KRIT1, MAP2K1, MAP2K7, MAP3K3, MTOR, NRAS, PIK3CA, PIK3R1, PIK3R2, PTEN, RASA1, SMAD4, SMAD5, TEK, TSC1, and TSC2*)

<u>Suppl Fig 1</u> : Control Cortex epileptogenicity is not mediated by a mTOR dependent chloride co transporter deregulation

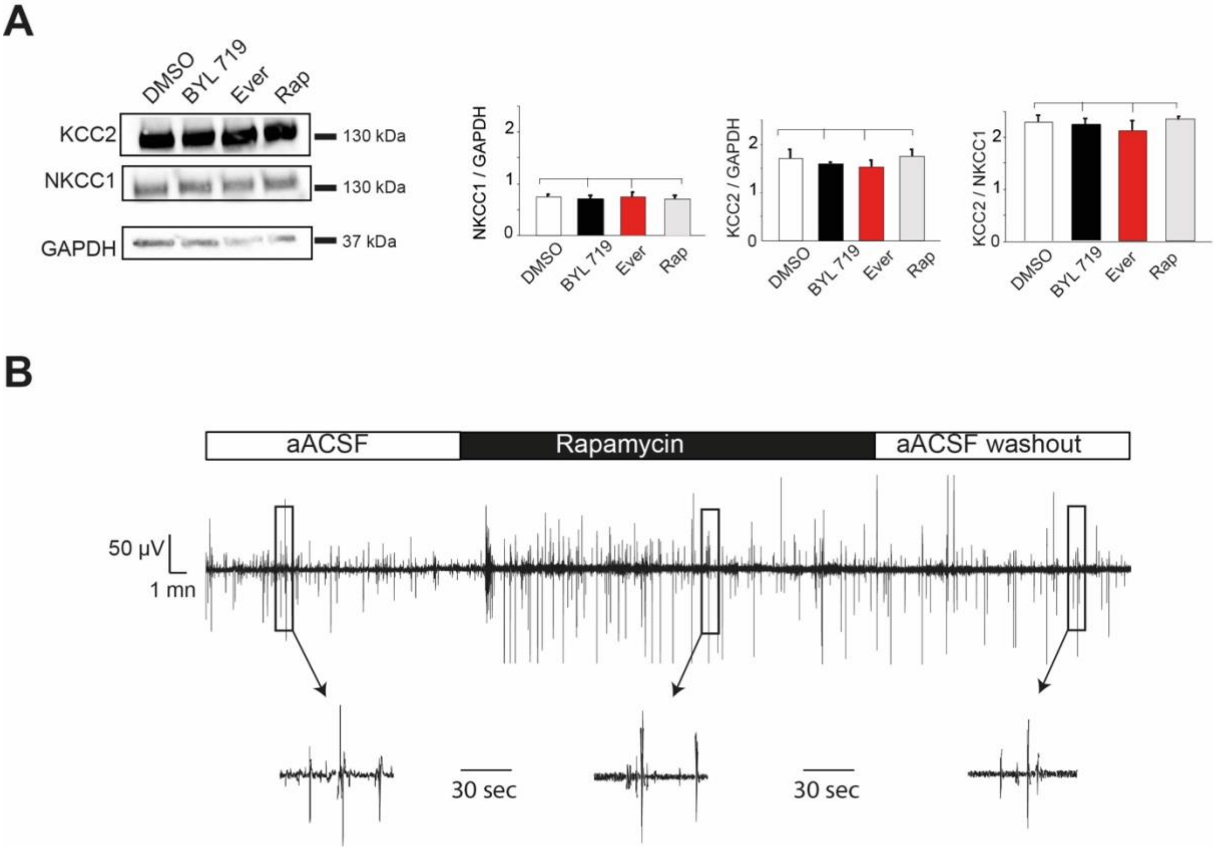

A/ Protein expression of KCC2 and NKCC1 in Control Human Brains. Western blots. quantifications of KCC2 and NKCC1. n = 4 western blots and N = 3 controls for both. KCC2 and NKCC1 expression levels are unchanged in all 3 control brains under BYL719, Everolimus and Rapamycin treatment compared to DMSO. The KCC2/NKCC1 ratio is not modified under treatments.

B/ MEA recordings shows spontaneous IID that are not suppressed by Rapamycin. Control Cortex from the left temporal pole resected during surgery for a ganglioglioma in the amygdala.

Suppl Fig 2: Time Frequency analysis at Low Frequencies (0.1-6Hz)

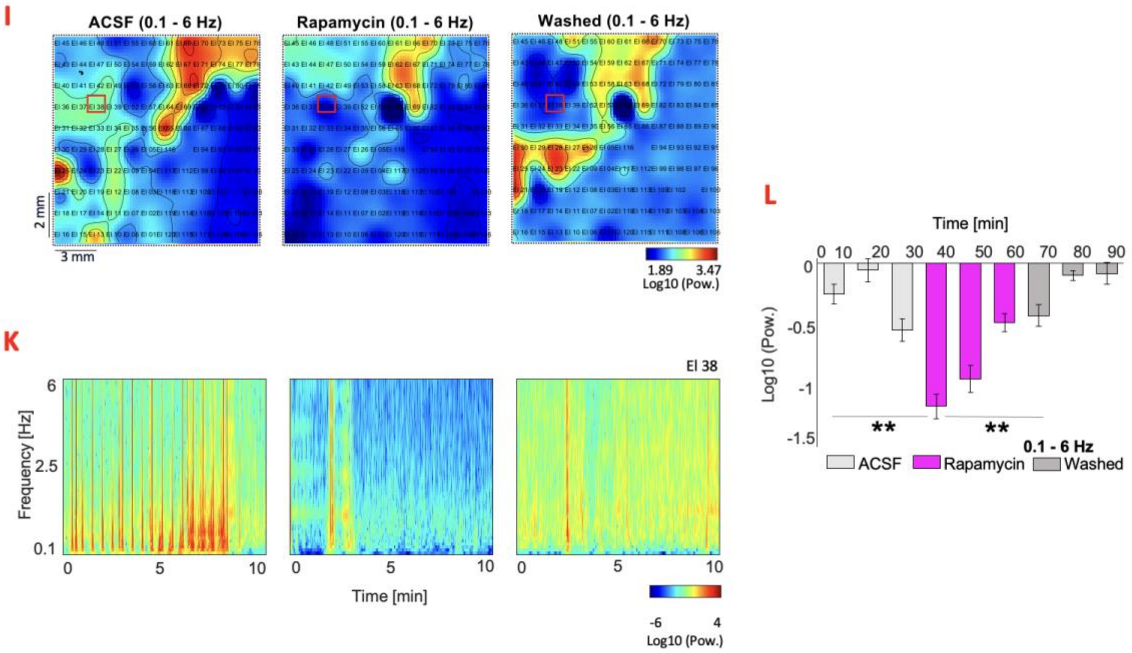

I: Heatmap examples for ACSF, Rapamycin and Washed, over the time-frequency window. The heatmaps use one value (Log10 (Pow.)) for each electrode, considering the whole distribution of the IID on the 120 contacts of the MEA grid. Red square indicates the active electrode that is further illustrated in *Panel K*. In ACSF, we observed a power distribution pattern, possibly associated with spreading, similar to that seen in the 2 – 40 Hz frequency range heatmaps. This pattern is suppressed in Rapamycin, but reactivated in Washed.

K: Time-resolved superlet spectra for the active electrode framed above in *Panel I* (ACSF, Rapamycin and Washed). The spectra illustrate a pattern of rhythmic oscillations, mostly suppressed in Rapamycin, and power reactivation in Washed.

L: Bar graphs representing continuous series of averages over a 10-minute interval each, for ACSF, Rapamycin, and Washed. Error bars = s.e.m. **=p<0.01

Suppl Fig 3: Western Blots

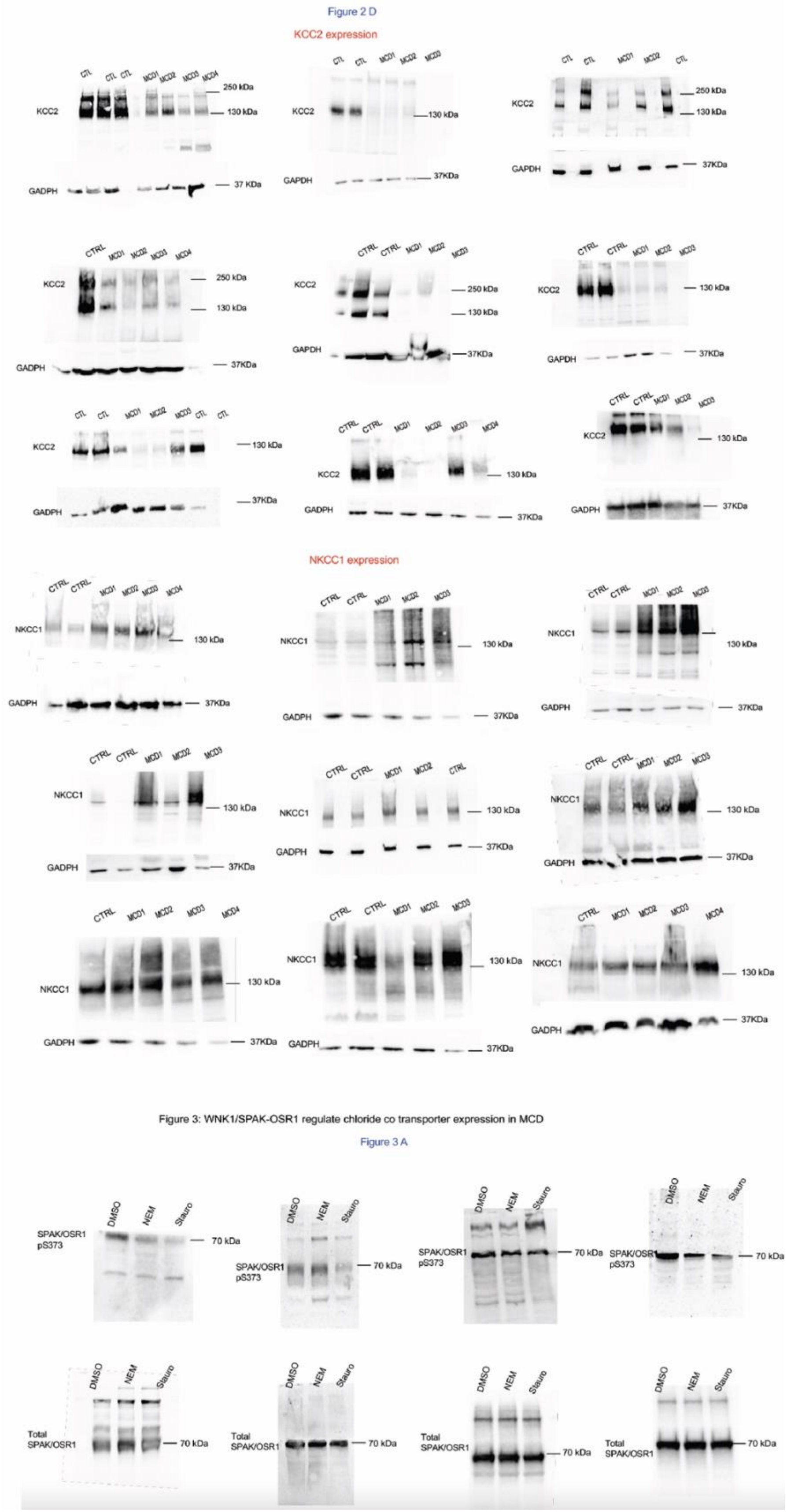

Figure 3: WNK1/SPAK-OSR1 regulate chloride co transporter expression in MCD

Figure 3 B

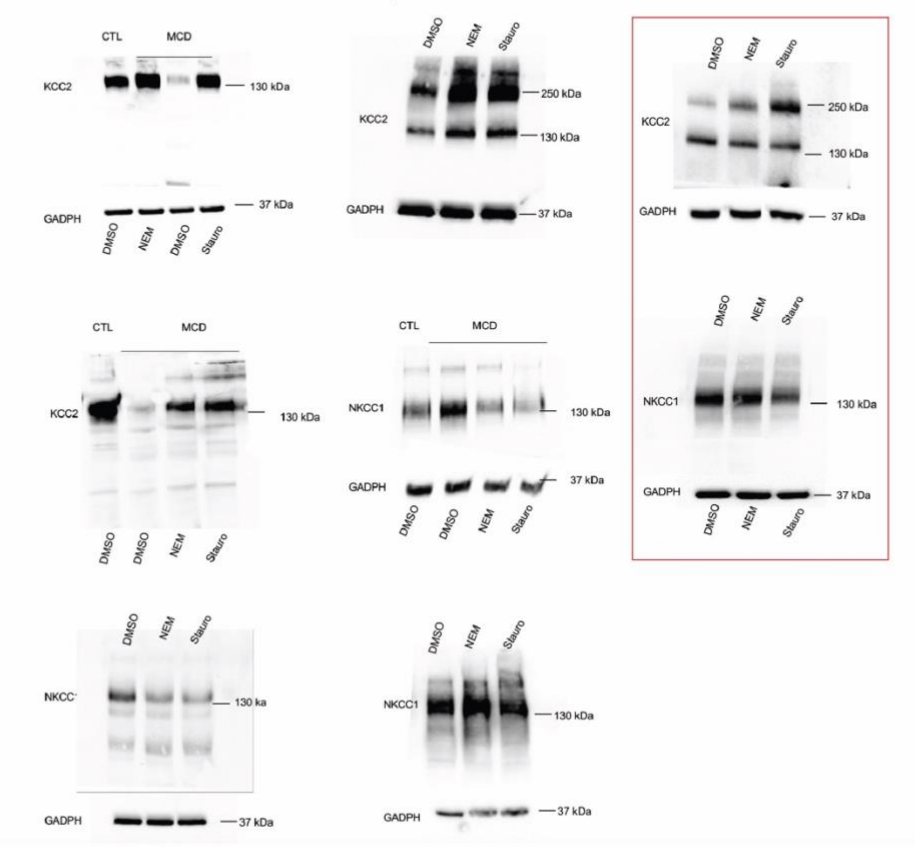

Figure 4: phosphorylation cascades underlying mTOR and CCC interactions.

Figure 4 A

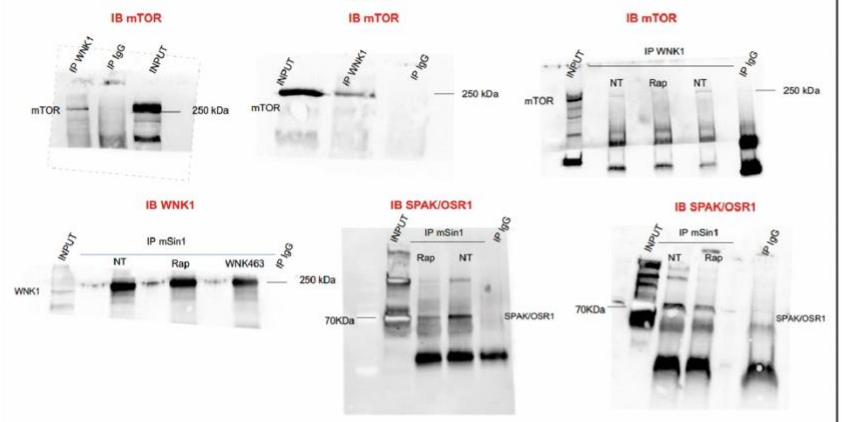

Figure 4: phosphorylation cascades underlying mTOR and CCC interactions.

Figure 4B left panel

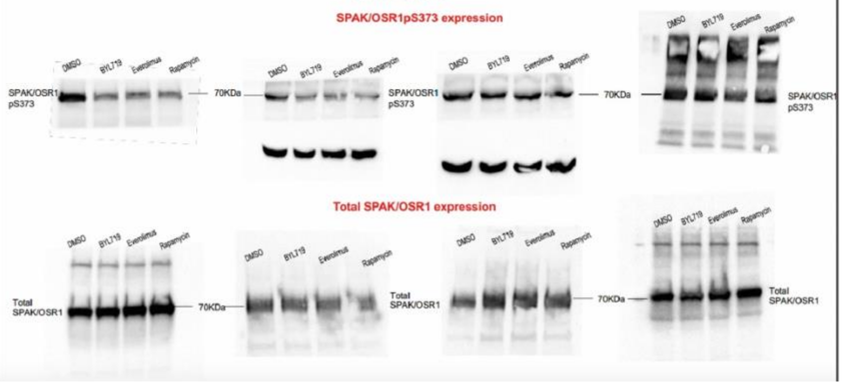

Figure 4: phosphorylation cascades underlying mTOR and CCC interactions.

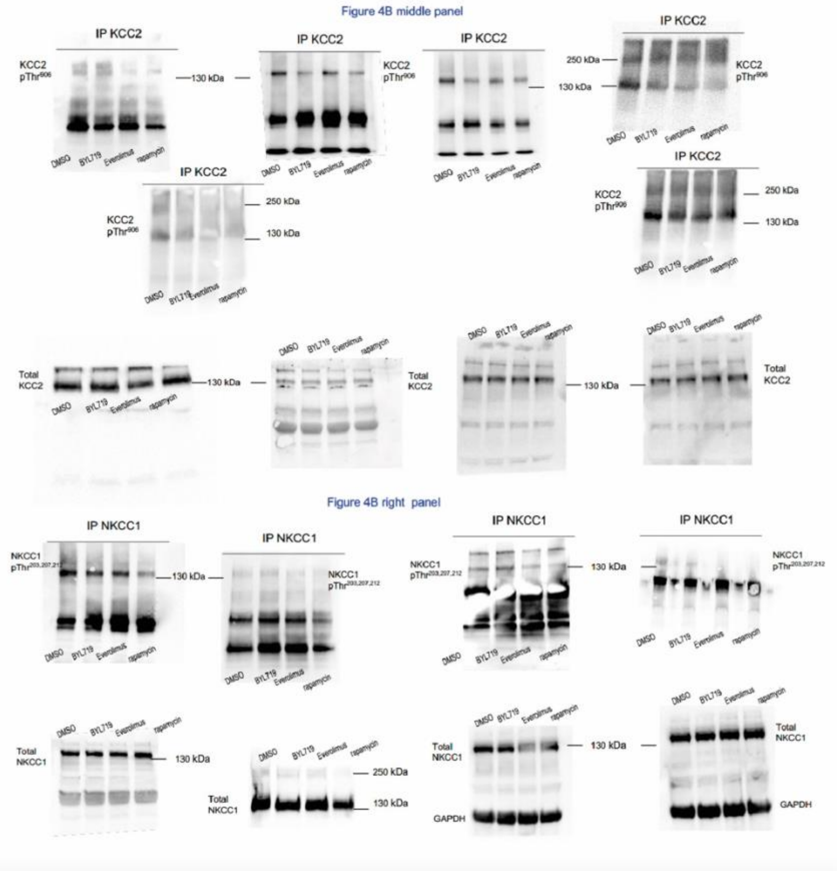

Figure 4C

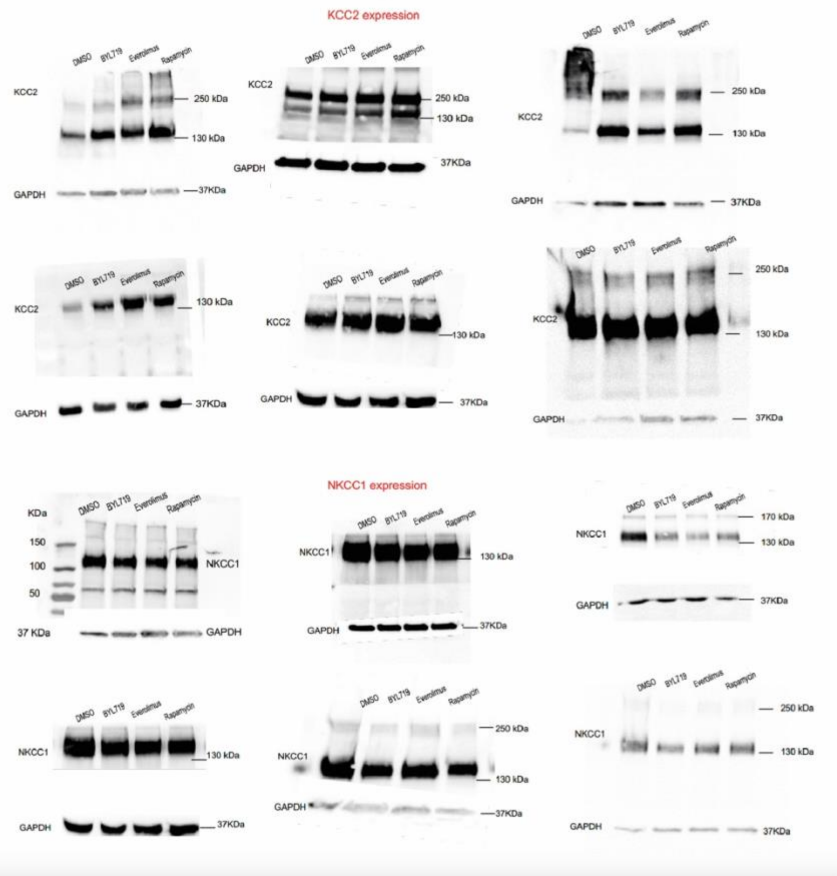

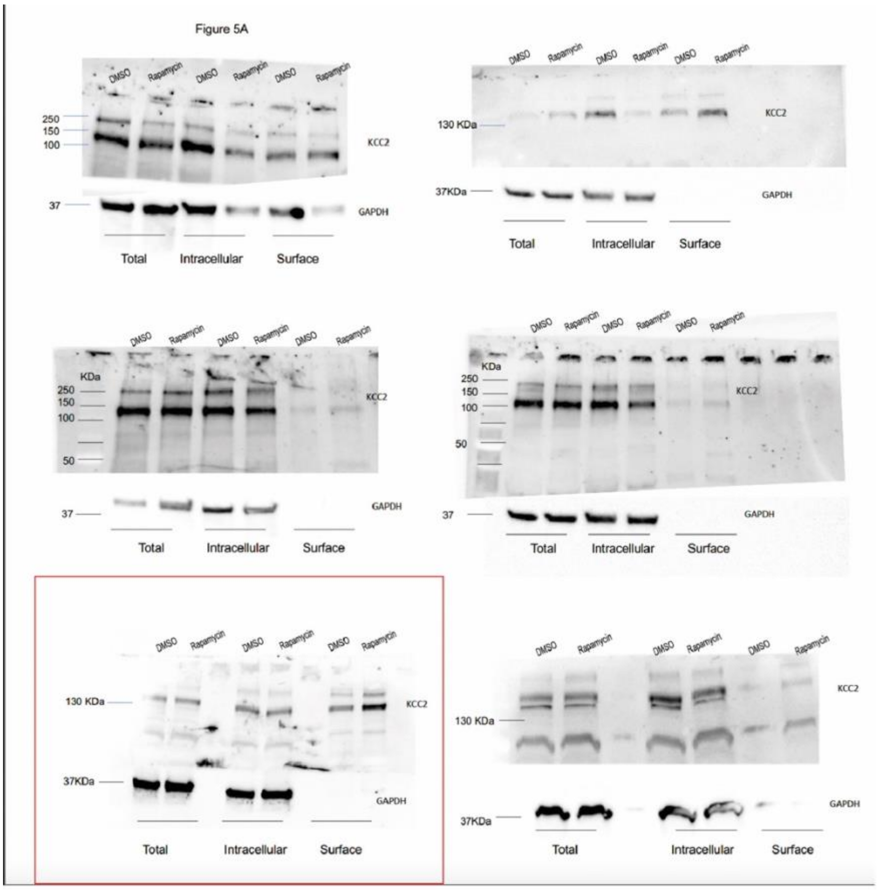
Figure 5A
DMSO
Rapamycin
250
150
100
37
KCC2
GAPDH
130 KDa
37KDa
KDa
50
Total
Intracellular
Surface

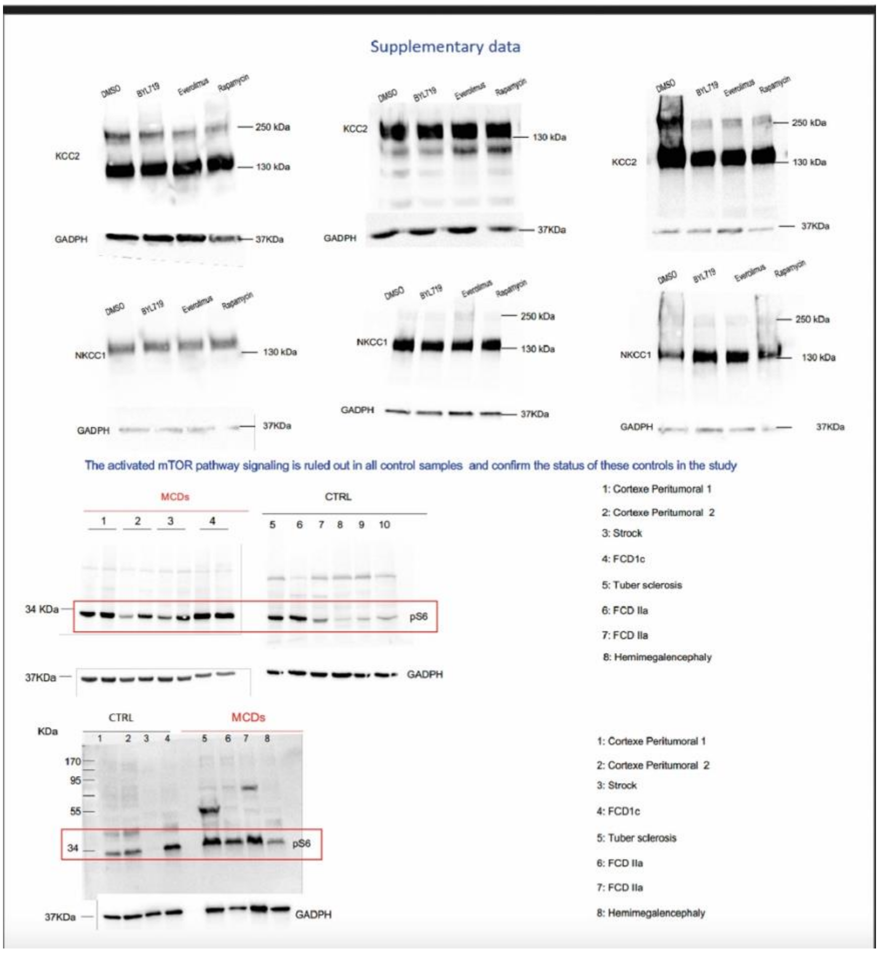
Supplementary data
DMSO
BYL719
Everolimus
Rapamycin
KCC2
NKCC1
GADPH
250 kDa
130 kDa
37KDa
The activated mTOR pathway signaling is ruled out in all control samples and confirm the status of these controls in the study
MCDs
CTRL
34 KDa
pS6
GADPH
KDa
170
95
55
34
1: Cortexe Peritumoral 1
2: Cortexe Peritumoral 2
3: Strock
4: FCD1c
5: Tuber sclerosis
6: FCD IIa
7: FCD IIa
8: Hemimegalencephaly

Table: Patient characteristics

Patient / Sex / Age at surgery	AEDs at time of surgery N / name	Surgery	Localization of sample	Histology	F.Up ILAE	Molecular Diagnosis	Slices N Ephy WB
**MTOR related surgeries**							
1- M - 2.3yo	3 / Carbamazepine-Vigabatrine-Topiramate	Left sided Hemispherotomy	Central region	Hemimegalencephaly	26m I		
2- F - 3.1yo	2/ Carbamazepine-vigabatrine	Right Sided Focal Resection	Operculo Insular	FCD IIb	26m I		MEA WB
3- M- 0.9 yo	2/ Lacosamide-Sodium Valproate	Right sided Occipital lobectomy	Occipital	FCD IIa	26m I		WB
4- M - 6.5 yo	4/ Vigabatrine-Lacosamide-Topiramate-Clobazam	Left sided Frontal lobectomy and insular resection after SEEG recording	Middle frontal gyrus	FCD IIa	22m III	MTOR A2532T 8%	MEA
5- M - 0.7	2/ Vigabatrine-Topiramate	Right sided Hemispherotomy	Central region	Hemimegalencephaly	22m IV		MEA WB
6- F – 3 yo	3 / Carbamazepine-Vigabatrine-Topiramate	Resection of 2 tubers after SEEG recording	Superior frontal Gyrus	Tuber	18m I	Sporadic TSC2 mutation (germline) PIK3CA R88Q 1%	MEA WB

7 – M - 1.7 yo	3/ Vigabatrine-Topiramate-levetiracetam	Right sided Parietal Focal Resection	Superior parietal lobule	FCD IIa	17m I	PIK3CA R93Q 2%	MEA WB
8 -M - 0.5 yo	3/ Vigabatrine - Topiramate-Tegretol	Left sided Temporal Lobectomy	Temporal	Tuber	15m I	Sporadic TSC2mutation	
9- F – 3.8yo	2/ Carbamazepine - Levetiracetam	Left sided Hemispherotomy	Central Region	Hemimegalencephaly	15m I	PIK3CA E542K 14%	MEA WB
10-M – 4.7 yo	2/ Topiramate - Lacosamide	Left Sided Frontal Focal Resection	Middle Frontal Sulcus	FCD IIb and focal neuronal Lipofuscinosis	14m I		MEA WB
11- F – 18.5	2/ Carbamazepine /Topiramate	Left Sided Frontal Focal Resection	Middle Frontal Sulcus	FCD IIb	12m I	MTOR S2215Y 2%	MEA WB
12- M – 6.3 yo	3/ Vigabatrine – Carbamazepine-Stiripentol	Right Sided Anterior Operculo Insular Resection after SEEG recording	Inferior frontal Gyrus	FCD IIb	12 I	TSC2 R1743Q 5%	MEA WB
13- M – 14.7 yo	1/ Lacosamide + Chlorpromazine-Fluoxetine	Left sided Frontal focal resection	Frontal pole	FCD IIb	11m I	SMAD4 R135* 3%	Biotinylation
14- M – 8.1 yo	4/ Sodium Valproate-Oxcarbazepine-Perampanel-Clobazam	Right sided Temporal Pole Resection after SEEG recording	Temporal Pole	FCD IIa	12m I		MEA
15- F – 1.08 yo	4/ Phenobarbital – Sodium	Left Sided Hemispherotomy	Central Region	Hemimegalencephaly	11m I		MEA

	Valproate-<br>Levetiracetam-<br>Clobazam<br>+ Ketogenic Diet						
16- F – 0.5 yo	4/ Vigabatrine – Sodium Valproate-Topiramate-Clonazepam	Right Sided Hemispherotomy	Central Region	Hemimagalencephaly	10m<br>I	MTOR C1534F 2%	MEA
17- M – 6.7 yo	2/ Sodium Valproate - Lamotrigine	Left Sided Frontal focal resection	Superior frontal sulcus	FCD IIb	9m<br>I		WB
18- M – 1.6 yo	3/ Vigabatrine – Sodium Valproate - Topiramate	Right Sided Posterior Quadrant Resection	Temporal	Tuber	7m<br>I	Germline: Sporadic TSC2 (deletion exon 1-42) and PKD1 mutation (deletion exon 35,44)<br>Somatic: PIK3CA R4* 2% VOUS	MEA<br>WB
19-M- 13.3 yo	3/ Sodium Valproate – Carbamazepine - Levetiracetam	Right Sided Parietal Resection after SEEG recording	Superior Parietal Lobule	FCD IIa	6m<br>I		MEA<br>WB

20- F – 10.9 yo	2/ Levetiracetam - Oxcarbazepine	Left Sided Temporal Resection after SEEG resection	Collateral Sulcus	FCD IIa and focal neuronal Lipofuscinosis	5m I		WB
21-F - 10 yo	2/ Vigabatril - Lamotrigine	Right sided Frontal Tuber resection after SEEG recording	Inferior frontal Gyrus	Tuber	5m IV	Sporadic TSC2 mutation	WB
22- F – 18.8 yo	2/ Lamotrigine - Carbamazepine	Right Sided Frontal Resection after SEEG recording	Orbito frontal gyri	FCD IIA	3m I		MEA WB
23- M – 2.6 yo	2/ Vigabatril - Topiramate	Left Sided Hemispherotomy	Central Region	Hemimegalencephaly	4m I	PIK3CA G106V 14%	WB
24-M – 8.9 yo	2/ Vigabatril - Tegretol	Left Sided Frontal Resection after SEEG recording	Inferior Frontal Gyrus	Tuber	4m I	Sporadic TSC2	MEA
25- M – 7.6yo	3/ Oxcarbazepine – Topiramate - Lacosamide	Left Sided Frontal focal resection	Superior frontal sulcus	FCD IIa	2m I		Biotinylation
**"CONTROL" , non mTOR CASES**							
26- F – 12.5 yo	3/ Sodium Valproate – Carbamazepine- Lamotrigine	Right Sided Hemispherotomy	Temporal Pole	Rasmussen	17m I		WB
27- F – 9.8 yo	5/ Sodium Valproate – Lamotrigin – Levetiracetam –	Right Sided Hemispherotomy	Central region	Rasmussen	21m I		MEA WB

	Perampanel-clonazepam						
28- M – 4.7	3/ Sodium Valproate – Lamotrigine - Levetiracetam	Right Sided Hemispherotomy	Central region	Rasmussen	15m<br>I		MEA<br>WB
29- F – 7.02	3/ Sodium Valproate – Lamotrigine - Clobazam	Left Sided Hemispherotomy	Central region	Stroke	24m<br>I		WB
30- M – 5.6	4/ Sodium Valproate – Lamotrigine – Rufinamide - canabidiol	Left Sided Hemispherotomy	Central region	Stroke	22m<br>I		MEA
31- M – 8.7yo	1/ Levetiracetam	Left Sided AVM resection	Frontal Pole	Cortex with Gliosis around AVM	19m<br>I		WB
32- F- 7.1	2/ Sodium Valproate - Oxcarbazepine	Left Sided Lesionnectomy	Occipital pole	Cortex with around Cavernoma	9m<br>I		
33- F – 5.1yo	2/ Sodium Valproate - Lacosamide	Left Sided Temporal lobectomy for a ganglioglioma	Temporal pole	Peri tumoral cortex with epilepsy associated unspecific abnormalities	19m<br>I		MEA
34- M – 7.1yo	2/ Carbamazepine - Clobazam	Right Sided Lesionectomy for a DNET	Parietal	Peri tumoral cortex with epilepsy associated unspecific abnormalities	18m<br>I		WB

35- F - 15.1 yo	1/ Carbamazepine	Right Sided Lesionectomy for a DNET	Temporal	Peri tumoral cortex with epilepsy associated unspecific abnormalities	15m<br>I		MEA<br>WB
